# A large structural variant collection in Holstein cattle and associated database for variant discovery, characterization, and application

**DOI:** 10.1186/s12864-024-10812-2

**Published:** 2024-09-30

**Authors:** Jason R. Grant, Emily K. Herman, Lael D. Barlow, Filippo Miglior, Flavio S. Schenkel, Christine F. Baes, Paul Stothard

**Affiliations:** 1 Agricultural, Food & Nutritional Science, University of Alberta, Edmonton, AB T6G 2P5 Canada; 2 Centre for Genetic Improvement of Livestock, Department of Animal Biosciences, University of Guelph, Guelph, ON Canada; 3 Lactanet, Guelph, ON Canada; 4 Institute of Genetics, Vetsuisse Faculty, University of Bern, Bern, Switzerland

**Keywords:** Cattle, Holstein, Structural variants, SNPs, Indels, Visualization, Database

## Abstract

**Background:**

Structural variants (SVs) such as deletions, duplications, and insertions are known to contribute to phenotypic variation but remain challenging to identify and genotype. A more complete, accessible, and assessable collection of SVs will assist efforts to study SV function in cattle and to incorporate SV genotyping into animal evaluation.

**Results:**

In this work we produced a large and deeply characterized collection of SVs in Holstein cattle using two popular SV callers (Manta and Smoove) and publicly available Illumina whole-genome sequence (WGS) read sets from 310 samples (290 male, 20 female, mean 20X coverage). Manta and Smoove identified 31 K and 68 K SVs, respectively. In total the SVs cover 5% (Manta) and 6% (Smoove) of the reference genome, in contrast to the 1% impacted by SNPs and indels. SV genotypes from each caller were confirmed to accurately recapitulate animal relationships estimated using WGS SNP genotypes from the same dataset, with Manta genotypes outperforming Smoove, and deletions outperforming duplications. To support efforts to link the SVs to phenotypic variation, overlapping and tag SNPs were identified for each SV, using genotype sets extracted from the WGS results corresponding to two bovine SNP chips (BovineSNP50 and BovineHD). 9% (Manta) and 11% (Smoove) of the SVs were found to have overlapping BovineHD panel SNPs, while 21% (Manta) and 9% (Smoove) have BovineHD panel tag SNPs. A custom interactive database (https://svdb-dc.pslab.ca) containing the identified sequence variants with extensive annotations, gene feature information, and BAM file content for all SVs was created to enable the evaluation and prioritization of SVs for further study. Illustrative examples involving the genes *POPDC3*, *ORM1*, *G2E3*, *FANCI*, *TFB1M*, *FOXC2*, *N4BP2*, *GSTA3*, and *COPA* show how this resource can be used to find well-supported genic SVs, determine SV breakpoints, design genotyping approaches, and identify processed pseudogenes masquerading as deletions.

**Conclusions:**

The resources developed through this study can be used to explore sequence variation in Holstein cattle and to develop strategies for studying SVs of interest. The lack of overlapping and tag SNPs from commonly used SNP chips for most of the SVs suggests that other genotyping approaches will be needed (for example direct genotyping) to understand their potential contributions to phenotype. The included SV genotype assessments point to challenges in characterizing SVs, especially duplications, using short-read data and support ongoing efforts to better characterize cattle genomes through long-read sequencing. Lastly, the identification of previously known functional SVs and additional CDS-overlapping SVs supports the phenotypic relevance of this dataset.

**Supplementary Information:**

The online version contains supplementary material available at 10.1186/s12864-024-10812-2.

## Background

Whole-genome sequence (WGS) datasets continue to expand for animals including cattle and other livestock species, and studies characterizing thousands of individuals within a given species have been published [1–4]. Generating information on sequence variation (sites of variation and genotypes), which is a main outcome of these efforts is challenging due to the resources involved in acquiring the sequence data and due to complexities of data sharing, management, and processing. Moreover, these analyses don’t produce a final, complete understanding of the genome variation in a population but rather these efforts are ongoing, with updates to software, reference genomes, sequencing technology, and sample numbers expected to deliver additional or more accurate information.

Structural variation is generally defined as chromosomal genomic rearrangements greater than 50 bp, including changes in copy number (insertions, deletions, and duplications), orientation (inversions) and chromosomal translocations and fusions. Structural variation is less explored than single-nucleotide variation (SNPs and indels); however, it has been shown to be an important source of monogenic and complex trait variation in cattle [5]. The Polled Celtic variant, found in some hornless cattle, is a complex 202 bp indel [6], while an 80 kb duplication is responsible for a polled phenotype in Friesians [7]. The colour sidedness phenotype observed in Belgian blue and brown Swiss cattle is the result of two serial translocation events involving the *KIT* locus [8]. SVs have also been found to cause genetic defects, such as a 3.3 kb deletion in the *FANCI* gene that leads to brachyspina syndrome in Holstein cattle [9], and an embryonic lethal 138 kb deletion on chromosome 9 encompassing the *TFB1M* gene [10]. SVs have been reported to be associated with economically important traits in Chinese indigenous cattle, such as immunity, meat production or quality, and hair development [11] and may play a role in environmental adaptability in Mongolian and Hainan cattle [12]. Several studies have suggested a link between copy number variation and phenotypes such as resistance to gastrointestinal nematodes in Angus cattle [13–15], mastitis in dairy cows [16], postpartum feed intake and hoof health [17], and feed intake in Holstein cows [18], while large deletions have been observed in association with reduced female fertility in *Bos indicus* [19] and Nordic Red cattle [20].

Here we describe the processing of whole-genome sequences from 310 Holstein cattle downloaded from NCBI SRA to deliver an updated, high-quality collection of structural variant (SV), SNP, and indel sites and genotypes. Although we and others have previously identified variants of various types including SVs in large collections of animals [11, 17, 21–31], the availability of more samples and the opportunity to apply new analysis and visualization tools led us to undertake this effort. To increase the utility of the resulting variant collections we also created an interactive database (https://svdb-dc.pslab.ca) that allows read alignments for all 310 samples to be inspected for any SV of interest in the dataset. Tag SNPs from WGS and overlapping and tag SNPs from two bovine SNP chips are included with each SV entry, along with functional impact predictions and genome feature overlap information. This dataset and accompanying database can be used in ongoing efforts to link sequence variation to phenotypic differences in Holstein cattle.

## Methods

### Samples

Holstein cattle genome sequences were identified in NCBI SRA and downloaded (Additional file 1), with the additional requirement that the datasets were generated by paired-end whole genome sequencing on an Illumina instrument, with a minimum average read length of 75 bp. Samples with a minimum fold coverage of 10x were sought, and for samples with a predicted coverage greater than 40x reads were randomly sampled to a target depth of 30x. Following read alignment (see Variant identification and genotyping), per-chromosome read depth was calculated for each sample using mosdepth [32], and sample sex was inferred through X:autosome and Y:autosome read depth comparisons.

### Variant identification and genotyping

Sequence reads were trimmed using Trimmomatic v0.39 [33] and aligned to the *Bos taurus* reference genome ARS-UCD1.2 [34] augmented with the Y chromosome from assembly Btau5.0.1 using burrows-wheeler aligner v0.7.17 [35]. Base quality score recalibration was performed using GATK BaseRecalibrator and ApplyBQSR [36]. Potential variant sites were detected using GATK HaplotypeCaller, and the resulting GVCF files were jointly genotyped with GATK GenotypeGVCFs. SNPs and indels were hard-filtered using GATK VariantFiltration. SNPs were filtered if they met any of the following criteria: QualByDepth (QD) < 2, FisherStrand (FS) > 60, RMSMappingQuality (MQ) < 40, MappingQualityRankSumTest (MQRankSum) < -12.5, ReadPosRandSumTest (ReadPosRankSum) < -8. Indels were filtered if they met any of the following criteria: QualByDepth (QD) < 2, FisherStrand (FS) > 200, ReadPosRandSumTest (ReadPosRankSum) < -20. SVs were identified using Manta [37] and Smoove [38]. Both SV callers use joint calling of genotypes in order to use cohort information to improve genotyping accuracy and create a single set of genotype calls across samples. The following filters were applied to generate a final set of Smoove SVs based on the developer’s recommendations [38]: Mean Smoove Het Quality > 3 for all SVs, and DHFFC (fold change for the variant depth relative to flanking regions) < 0.7 for deletions and > 1.25 for duplications. Manta does not suggest parameters for downstream filtering. The MSHQ is the mean quality score across heterozygous samples for that variant, ranging from 1–4. The DHFFC filter discards SVs where the average coverage within the SV is too similar to that of the surrounding region, as this can indicate a false positive. Manta does not report MSHQ and DHFFC, as these are specific to Smoove and its component programs. It is possible to do further filtering of both files, as Manta and Smoove report FORMAT values related to genotype quality, depth, and paired read support, but in the interest of generating an inclusive set of SVs, we opted not to do this. SVs larger than 0.5 Mb were removed, as were variants on non-chromosomal contigs.

### SV characteristics and SV set overlap

Graphical and text summaries of SV characteristics were generated from SV VCF files using bcftools [39], vcftools [40], the pysam [41, 42] Python package, and the karyoploteR [43] R package. For the calculation of total and percent affected bases, SNP loci were counted as a single base, indels and SV insertions as the absolute difference between the reference allele and first alternative allele, and SV deletions and SV duplications as the length of the reference allele. A set of SVs detected by both Manta and Smoove (“shared SVs”) was generated using a Python script that uses pysam [44]. The following criteria were used to identify shared SVs: the SV type was the same; the SV boundaries overlapped such that the overlapping portion represented at least 90% of the length of each SV; at least 90% of the genotypes matched; and there were no opposing homozygous genotypes. When plotting characteristics of the shared set or when using the genotypes of the shared set to estimate animal relationships, the Manta-supplied genotypes were used unless stated otherwise.

### SV genotype evaluation

Autosomal SV genotypes from Manta (*n* = 30,112; mean per-site missing rate = 0.000), Smoove (*n* = 65,687; mean per-site missing rate = 0.003), and both callers (shared SVs; *n* = 8,443; mean per-site missing rate = 0.000) were each used to estimate sample relationships using the KING kinship coefficient estimation [45] as implemented in vcftools and run using the –relatedness2 option. For each pair of samples, the kinship coefficient was also estimated using SNPs. Due to the large number of SNPs identified, a single random sample of 20,000 biallelic sites with missing rate less than or equal to 0.1 was used in place of the full SNP set. For each animal pair the relationship inferred from SVs was plotted against that obtained using SNPs, and the Pearson correlation was calculated using results for all sample pairs. Because loci number can affect the KING results [45], an additional random sampling approach was used where the correlation was calculated between the SV and SNP estimates for each of 100 random samples (with replacement) of 2,000 SV loci. An indel vs SNP comparison was also performed, using a random sample of 20,000 indels (biallelic; missing rate <  = 0.1) as the set from which 100 samples of size 2,000 were drawn. The distributions of correlations were then plotted for all SV sets and the indel set.

### SV overlap with SNP chip markers

The positions of BovineSNP50 (v3; 53,218 loci) and BovineHD (777,962 loci) SNP chip (Illumina, San Diego, CA, USA) loci were determined on the study genome assembly using genotype_conversion_file_builder [46]. These locations were then compared to those of the SVs using the pysam and pandas [47, 48] Python packages to identify overlapping SNPs for each SV (i.e. SNPs with positions located within the SV region).

### Linkage disequilibrium between SVs and SNPs

The genotypes for SNPs on the BovineSNP50 and BovineHD SNP chips were extracted from the study SNP VCF file using position and allele information generated using genotype_conversion_file_builder. A custom Python script was used to calculate the squared correlation coefficient *r*^2^ between SVs and SNP chip loci, and between SVs and WGS SNPs, encoded as 0, 1 and 2 to represent the number of non-reference alleles. The *r*^2^ values are equivalent to those obtained using the vcftools –geno-r2 option [40]. For each SV, SNPs were examined beginning on either side of the SV and extending up to 10 kb away. SNPs with *r*^2^ >  = 0.8 were recorded as tag SNPs; once five tag SNPs were identified no further SNPs were considered for that SV.

### Variant annotation

Reference SNP and indel IDs, or rs IDs, were assigned to SNPs and indels using bcftools annotate [39] with reference SNP and indel data (*Bos taurus* release 5 for ARS-UCD-1.2 and Btau_5.0.1) downloaded from the European Variant Archive (EVA) [49]. Ensembl VEP version 105 [24] was used to make functional impact predictions for SVs, SNPs, and indels. VEP was run using the –pick option, which reports one set of annotations per variant. To obtain a complete list of genes with overlapping exons or coding sequences (CDS) for each SV, bedtools intersect [50] was used to compare SV sites to genome features downloaded from Ensembl (Bos_taurus.ARS-UCD1.2.105.chr.gtf.gz for autosomes, the X chromosome, and the mitochondrial genome) and NCBI (GCF_000003205.7_Btau_5.0.1_genomic.gff.gz for the Y chromosome). An overlap of at least 10 bp was required for the overlap to be recorded, to reduce the number of overlaps caused by processed pseudogenes, which are mRNA-derived copies of genes inserted in the genome that can lead to artifactual deletions spanning introns with, in some cases, exon overlap.

### Structural variant database

SV, SNP, and indel information from this study are available with raw read alignments and analysis results through a custom database called the Structural Variant Database for Dairy Cattle (SVDB-DC). SVDB-DC is freely accessible at https://svdb-dc.pslab.ca and is hosted on Digital Research Alliance of Canada cloud resources [51]. SVDB-DC is written in JavaScript and uses the React framework [52] for UI components and JBrowse 2 [53] for the genome viewer. SVDB-DC includes the VEP-annotated SVs, SNPs, and indels as well as BAM file content for genome segments that overlap with the SVs (plus 50% of the length of the SV on either side), from all 310 samples. Searchable “SV cards” provide extracted content from the VCF files (e.g. allele frequency information, genotype counts, Hardy–Weinberg equilibrium (HWE) information, impact consequences), as well as the results from other analyses not captured in the VCF file (overlapping SNPs, tag SNPs, whether the SV was identified by both SV callers, genome feature overlap). When an SV is viewed in the database, read alignments are automatically loaded for a random selection of samples from each observed genotype class.

## Results

### Samples

**Fig. 1:**
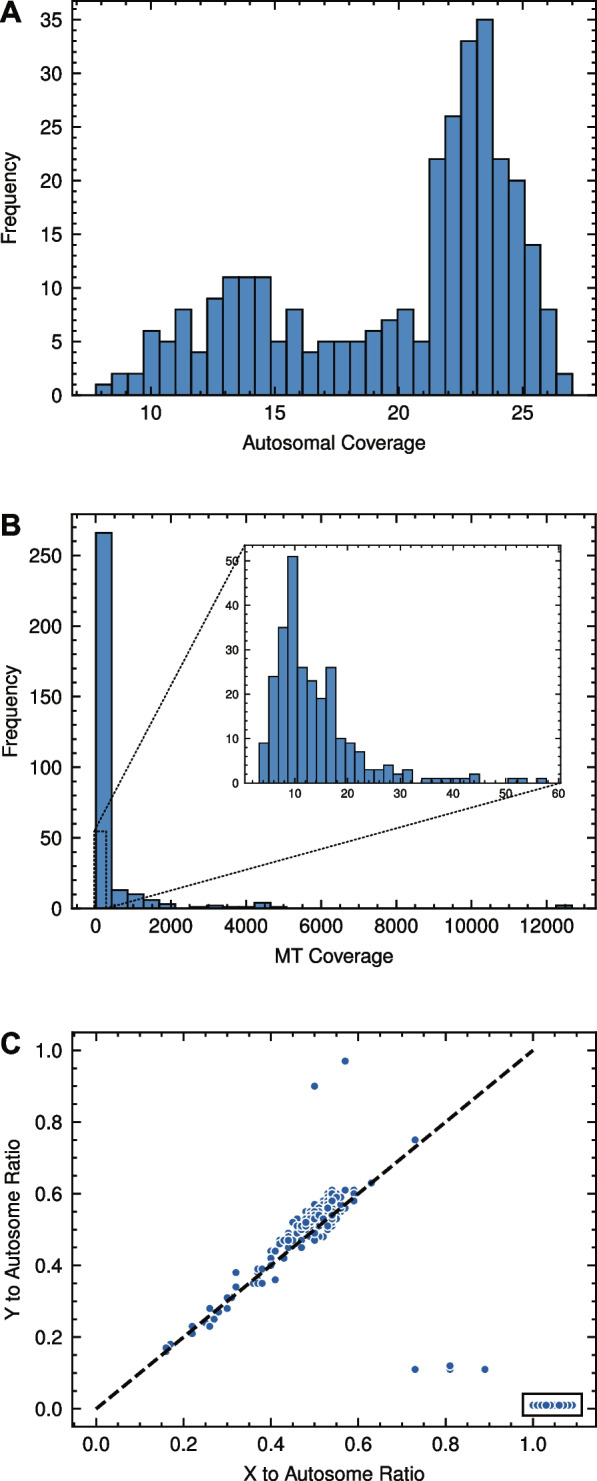
Autosomal fold coverage and sex chromosome to autosome fold coverage ratios for the 310 Holstein samples processed in this study. **A** Histogram of autosome fold coverage. **B** Histogram of MT genome coverage with an inset plot showing for 0-to-60-fold coverage (**C**) Sex assignment of samples based on a scatter plot of X to autosome ratio vs Y to autosome ratio. Boxed samples were classified as female

The 310 Holstein samples yielded between 8- and 27-fold autosome coverage (Additional file 1), with a mean autosome coverage of 20-fold (Fig. 1A). As MT variants were assessed we examined the coverage of the MT genome. A much larger range of values was obtained (Fig. 1B) possibly reflecting different sample sources. For sex assignment, autosome to sex chromosome ratios were visualized (Fig. 1C), leading to the assignment of 290 samples as male and 20 samples as female. The inferred sex matched the sex stated in the NCBI BioSample record in all 208 cases where sex information was provided (197 male and 11 female). Interestingly, two of the male samples, SRS3416799 and SRS7490417, produced Y:autosome ratios close to 1 with X:autosome ratios of near 0.5, which is consistent with an XYY genotype or XY/XYY mosaicism, but further work (e.g. karyotyping) would be required to confirm this to be the cause. Four male samples showed coverage patterns intermediate between female and male (ERS2600184, ERS2600232, ERS2600263, ERS2600264). Our inferred sex (male) for these samples is consistent with the BioProject description (ID PRJEB27379) which states that popular sires were sequenced. The reason for the unusual coverage ratios for these samples is not known.

### Variant identification, SV characteristics and SV set overlap

**Fig. 2:**
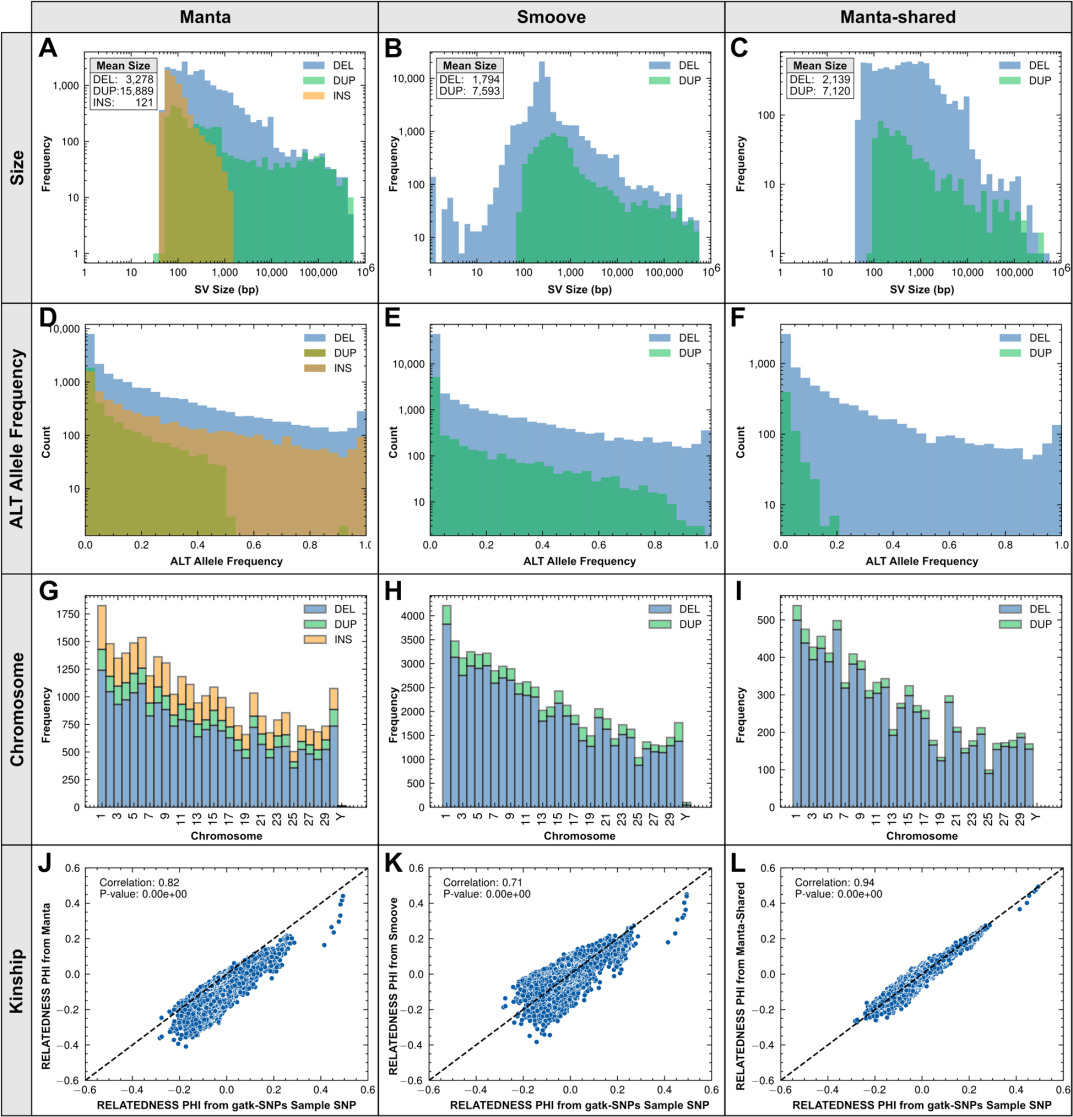
Structural variant characteristics. **A**-**C** Size distributions by type. **D**-**F** Alternative allele frequency distributions by type. **G**-**I** Chromosomal distributions by type. **J**-**L** KING metric correlation plots. Each point represents a pair of samples and is plotted based on the kinship coefficient estimated from SVs (vertical axis) and from SNPs (horizontal axis). Characteristics are provided for the Manta (ADGJ), Smoove (BEHK) and Manta-shared (CFIL) datasets

Among the 310 study samples, GATK identified approximately 20 M SNPs and 3 M indels and Manta and Smoove returned 31 K and 68 K bi-allelic SVs, respectively (Table 1). The potential functional importance of SVs is reflected in this dataset by the total size of the affected genome regions relative to that affected by SNPs (Table 2). Comparison of the size distributions of the variants identified by Manta (Fig. 2A) and Smoove (Fig. 2B) highlights differences between the program results. For example, Manta did not report any deletions or insertions less than 50 bp in length. Curiously, Smoove reported several (140) deletions 1 bp in length (note the bar on the left side of Fig. 2B). These were removed during annotation (VEP flagged them as errors) and are not included in the final SV database. Alternative allele frequency distributions for Manta (Fig. 2D) and Smoove (Fig. 2E) appear to be similar for deletions but not duplications, with a pronounced absence of mid to higher frequency duplications in the Manta results. The cause of this absence is unknown. To visualize the distribution of SVs on the genome, Manta SV, Smoove SV, and gene locations and densities were plotted on the reference assembly using karyoploteR (see Additional file 2). The plot shows that both callers identify variants throughout the genome, and that there are many regions where both callers report a high density of SVs. This pattern is assumed to be influenced both by the true SV distribution and by the limitations of short-read assessment of repetitive or low-complexity regions of the genome.

**Table 1:** Number of each variant type detected in the Holstein dataset by Manta, Smoove and GATK

Type	Manta	Smoove	Shared^a^	GATK
DEL	21,566	60,500	8029	-
DUP	3405	7050	583	-
INS	6228	-	-	-
INDEL	-	-	-	3,281,177
SNP	-	-	-	19,857,394
ALL	31,199	67,550	8612	23,138,571

^a^Shared represents the counts of SVs detected by both Manta and Smoove

**Table 2:** Number and percentage of reference genome bases covered by variants detected with Manta, Smoove and GATK

Type	Manta	Smoove	GATK
DEL	70,723,108 (2.65%)	108,569,775 (4.06%)	-
DUP	54,106,751 (2.03%)	53,541,126 (2.00%)	-
INS	735,523 (0.03%)	-	-
INDEL	-	-	15,043,166 (0.56%)
SNP	-	-	19,857,394 (0.74%)
ALL	125,565,382 (4.70%)	162,110,901 (6.07%)	34,900,560 (1.31%)

Percentage (shown in brackets) of genome based on a genome (1–29, X, Y, MT) length of 2,671,711,444 bp (Bos taurus reference genome ARS-UCD1.2 augmented with the Y chromosome from assembly Btau5.0.1.)

### SV genotype evaluation

**Fig. 3:**
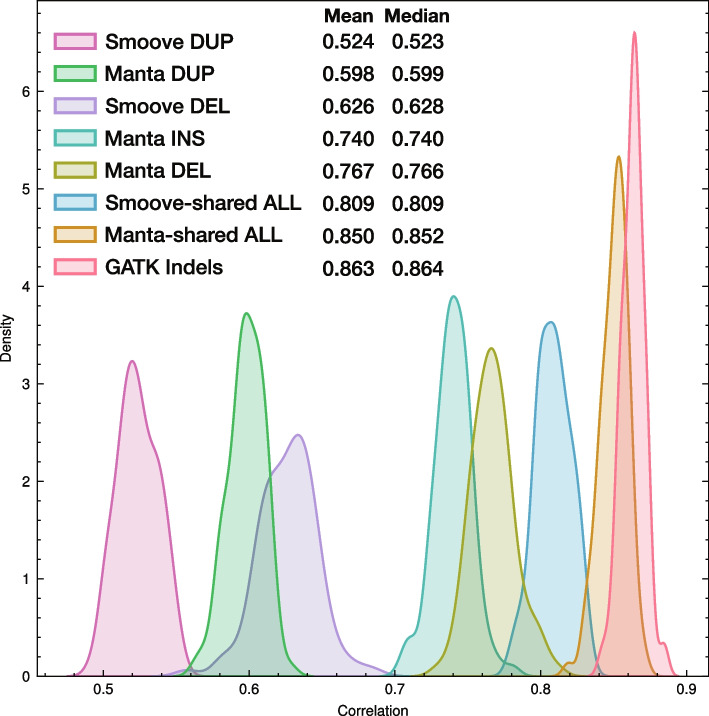
Distributions of correlations between kinship coefficients calculated for all pairs of animals using 100 random samples of 2,000 SVs or 2,000 indels and 20,000 SNPs. “Manta-shared ALL” consists of SV sites of all types identified by both Manta and Smoove, and with the Manta-supplied genotypes used in the analysis. “Smoove-shared ALL” consists of SV sites of all types identified by both Manta and Smoove, and with the Smoove-supplied genotypes used in the analysis. Mean and median values of correlations are provided in figure key

As a quality control step to assess SV genotype quality and to confirm sample correspondence across the various variant data sets, SV genotypes from Manta and Smoove were used to estimate relationships between each pair of animals in the dataset, using kinship coefficients estimated using KING [45]. The SVs from both programs and from the merged set produced kinship coefficients highly correlated to those from SNPs (Fig. 2JKL). To better compare the performance of the different SV types and programs in terms of the ability of genotypes to estimate animal relationships (as a proxy of genotype quality in the absence of knowledge of known SVs or trios), the analysis was repeated using random samples of variants (100 samples of 2,000 loci), to remove the effect of loci number on the results. GATK indel genotypes produced the highest correlation with SNPs, followed by the shared set of SVs (Fig. 3). Deletions outperformed duplications, and for a given SV type the Manta results outperformed those of Smoove (Fig. 3).

### Structural variant overlap with SNP chip markers

To gauge whether the discovered SVs can potentially be genotyped using SNP chip signal intensity-based approaches like those used by PennCNV [54] and QuantiSNP [55], overlapping SNPs (i.e. within SV boundaries) from the BovineSNP50 and BovineHD SNP chips were identified for each SV. This analysis was conducted for the SNP chip loci for which positions could be determined on the reference genome (53,124 loci from the BovineSNP50 chip and 776,694 loci from the BovineHD chip). For both the Manta and Smoove sets most of the SVs of a given type have no overlapping markers on the BovineSNP50 chip (Table 3). Even with the higher marker number of the BovineHD chip most of the identified SVs do not overlap with a marker (Table 3). There are more duplications with SNP overlaps than deletions with SNP overlaps, consistent with the larger size of duplications in the dataset (Manta duplications have a mean length of 15,889 bp vs 3,278 bp for deletions; Smoove duplications have a mean length of 7,593 bp vs 1,798 bp for deletions). In total, 946 BovineSNP50 panel markers overlapped with at least one SV identified by Manta, and 1,265 markers overlapped with at least one SV identified by Smoove. For the BovineHD SNP chip, 14,519 markers overlapped with Manta-identified SVs, and 20,518 markers overlapped with Smoove-identified SVs.

**Table 3:** Number and percentage of SV loci that overlap with markers on the BovineSNP50 and BovineHD SNP chips

Source	Type	BovineHD	BovineSNP50
Manta	DEL	2012 (9.33%)	376 (1.74%)
Manta	DUP	849 (24.93%)	283 (8.31%)
Manta	INS	0 (0%)	0 (0%)
Manta	ALL	2861 (9.17%)	659 (2.11%)
Smoove	DEL	6044 (9.99%)	715 (1.18%)
Smoove	DUP	1529 (21.69%)	302 (4.28%)
Smoove	All	7573 (11.21%)	1017 (1.51%)

Counts are of the number of SV loci with 1 or more SNP overlaps. Percentage is based on the total number of SVs for the given category in Table 1

### Structural variant LD with WGS and SNP chip markers

To examine the potential of detecting the effects of these SVs on phenotype through tag SNP genotyping, tag SNPs (*r*^2^ >  = 0.8 and within 10 kb) from the BovineSNP50 and BovineHD SNP chips and from the set of WGS SNPs were identified. This analysis was conducted for the SNP chip loci for which genotypes could be extracted from the SNP VCF file (49,115 loci from the BovineSNP50 chip and 667,612 loci from the BovineHD chip). For the Manta SVs, the best tagged variants are insertions followed by deletions and then duplications, whereas for Smoove, duplications are better tagged than deletions (Table 4). Overall, Manta variants are better tagged by the three SNP sources (49% by WGS, 21% by BovineHD, 4% by BovineSNP50) compared to Smoove variants (23% by WGS, 9% by BovineHD, 1% by BovineSNP50). Since the *r*^2^ results are influenced by genotype accuracy, the true proportion of SVs with SNPs meeting our tag SNP criteria could be higher.

**Table 4:** Number of SV loci with tag SNPs from the BovineSNP50 and BovineHD SNP chips and from the set of WGS SNPs

Source	Type	BovineHD	BovineSNP50	WGS
Manta	ALL	6636 (21.27%)	1161 (3.72%)	15,336 (49.16%)
Manta	DEL	4778 (22.16%)	850 (3.94%)	10,625 (49.27%)
Manta	DUP	61 (1.79%)	8 (0.23%)	707 (20.76%)
Manta	INS	1797 (28.85%)	303 (4.87%)	4004 (64.29%)
Smoove	ALL	5785 (8.56%)	981 (1.45%)	15,473 (22.91%)
Smoove	DEL	5603 (9.26%)	960 (1.59%)	12,460 (20.60%)
Smoove	DUP	182 (2.58%)	21 (0.30%)	3013 (42.74%)

Counts are the number of SV loci with 1 or more tag SNPs. Percentage is based on the total number of SVs for the given category in Table 1

### Variant annotation and prioritization

To assess the potential biological significance of the identified SNPs, indels, and SVs, functional consequence predictions (e.g. intron_variant, transcript_ablation, etc.) and impact categories (HIGH, MODERATE, LOW, MODIFIER) were obtained for all variants using Ensembl VEP [56]. For Manta and Smoove, 3.1% and 1.5% of the sites were annotated as having a predicted high impact, respectively (data not shown). VEP was run using the –pick option, which causes it to report a single block of annotation for each site chosen based on an ordered set of criteria that favors, for example, transcripts supported by stronger evidence. While this option helps to keep output files more manageable it necessarily omits some possible consequences for some variants (e.g. a deletion that spans multiple genes will have one of the genes reported). Moreover, we noticed inexplicably low impact predictions for some known SVs of high impact. For this reason, an additional feature overlap analysis was used to generate comprehensive lists of genes that overlap by exon or CDS for each SV. Manta and Smoove SVs were found to have exon overlap with 1,538 and 3,355 genes and CDS overlap with 1,214 and 2,624 genes, respectively (data not shown). SV information from Manta and Smoove including genotype summary information, VEP impacts, overlapping genes, overlapping panel SNPs, tag SNPs, and links to visualize read alignments is provided in Additional files 3 and 4 (respectively).

### The Structural Variant Database for Dairy Cattle (SVDB-DC)

**Fig. 4:**
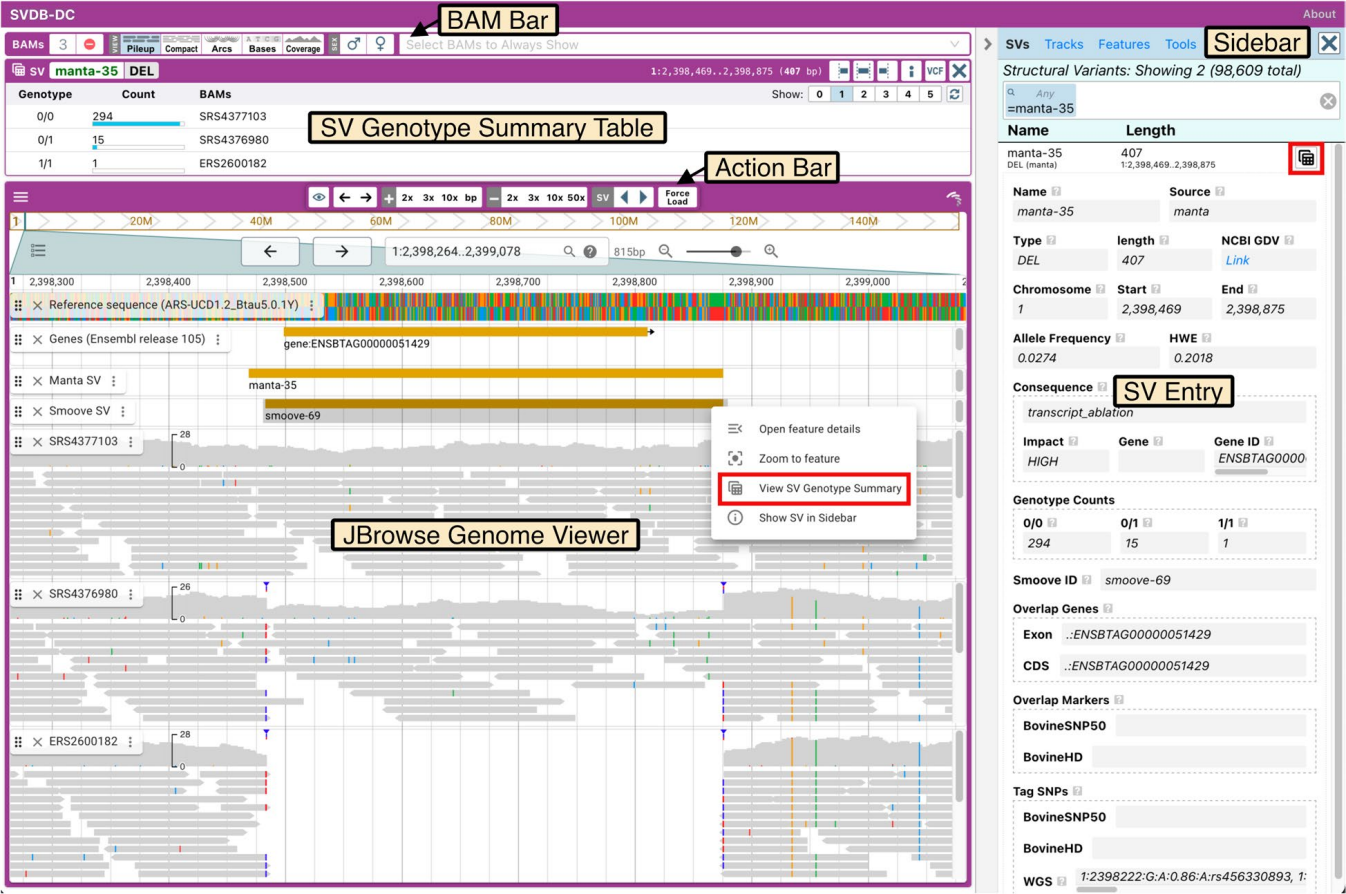
The Structural Variant Database for Dairy Cattle (SVDB-DC) interface. SVDB-DC consists of a JBrowse genome viewer with a sidebar on the right consisting of multiple panels (the SVs panel with search bar is shown). Clicking the table button (highlighted in red) in an SV entry in the SVs panel, or right clicking an SV in the genome browser and choosing “View SV Genotype Summary” (highlighted in red) displays an SV genotype summary table, which includes controls for choosing how many BAM tracks to show per genotype class. The BAM Bar contains buttons for adjusting how BAM tracks are displayed and for controlling which samples are displayed. The Action Bar is used to navigate within the genome browser

To assist with the sharing and evaluation of the results of this study, we created the Structural Variant Database for Dairy Cattle (SVDB-DC). The SVDB-DC user interface consists of two main areas, the genome viewer and sidebar. By default, the genome viewer shows the reference genome (ARS-UCD1.2_Btau5.0.1Y), genes (Ensembl release 105), GATK SNPs and Indels, and Manta and Smoove SVs (this study). In addition, BAM tracks are available for all 310 samples for visual inspection of any SV of interest in the dataset. The sidebar has tabbed panels for easy access to SVs, tracks, features, and tools. The SVs panel contains the list of all SVs found in this study and a powerful search interface that can be used to find SVs based on numerous search criteria, including location, Hardy–Weinberg equilibrium (HWE) p-value, alternative allele frequency (AF), VEP consequences, and VEP impact (Fig. 4). The Tracks panel displays a list of the tracks that have been added to JBrowse and includes buttons to minimize tracks, view track details, and add additional tracks. The Features panel is a filterable list of features currently visible in the JBrowse viewer. Lastly, the Tools panel provides buttons to open the currently visible map region in three external genome browsers: NCBI Genome Data Viewer [57], UCSC Genome Browser [58], and the Ensembl Genome Browser [59]. Using the NCBI Genome Data Viewer, for example, it is possible to load RNA-seq data sets from various tissues to assess whether there is evidence for the expression of a particular gene or exon located within the region.

The default JBrowse genome browser interface has been updated with additional custom components to improve usability when working with several alignment (BAM) tracks. Floating above the genome browser is an “Action Bar” with quick action buttons to pan, zoom and cycle through SVs. The Action Bar remains in place when scrolling through multiple tracks. While adding tracks is straightforward in JBrowse, customizing them one by one can be laborious. To streamline the addition and adjustment of BAM tracks, we implemented a “BAM Bar” above the JBrowse map (Fig. 4). The BAM Bar includes view options to change the display format of all the BAM tracks and features a searchable multi-select box for selecting specific BAMs to display on the map.

Representative BAM tracks for an SV of interest can be viewed by right clicking the SV in the genome browser and selecting “View SV Genotype Summary”, or, if viewing the SV in the SVs panel, by clicking the table button. These actions display an SV genotype summary table above the map that lists each observed genotype (e.g., 0/0, 0/1, 1/1), the number of samples per genotype in the full dataset, and the samples (BAM tracks) displayed for each genotype. Users can adjust the number of BAM tracks shown per genotype from zero to five. A refresh button is available to quickly load a new set of randomly chosen BAM files. Sample sex information in the database allows for the selection to be restricted to XX or XY animals, which is useful when studying sex-linked loci.

SVDB-DC supports a simple URL API for quickly accessing individual SVs. The URL format is https://svdb-dc.pslab.ca/?sv=SVID where SVID is the ID of the SV (e.g. manta-1234 or smoove-2345). This API automatically positions the genome browser to the SV region, displays the corresponding SV genotype summary table above the genome browser, and loads the textual SV details in the SV panel. Additional files 3 and 4 use this URL structure to provide a rapid means of visualizing SVs of interest in SVDB-DC.

Users wishing to perform additional analysis on the SVs (filtering for example) can download the corresponding VCF files from SVDB-DC using the Tracks panel. Clicking on the information button next to a track name in the Tracks panel opens an information window containing links to the relevant data files.

### Examining previously described SVs in SVDB-DC

**Fig. 5:**
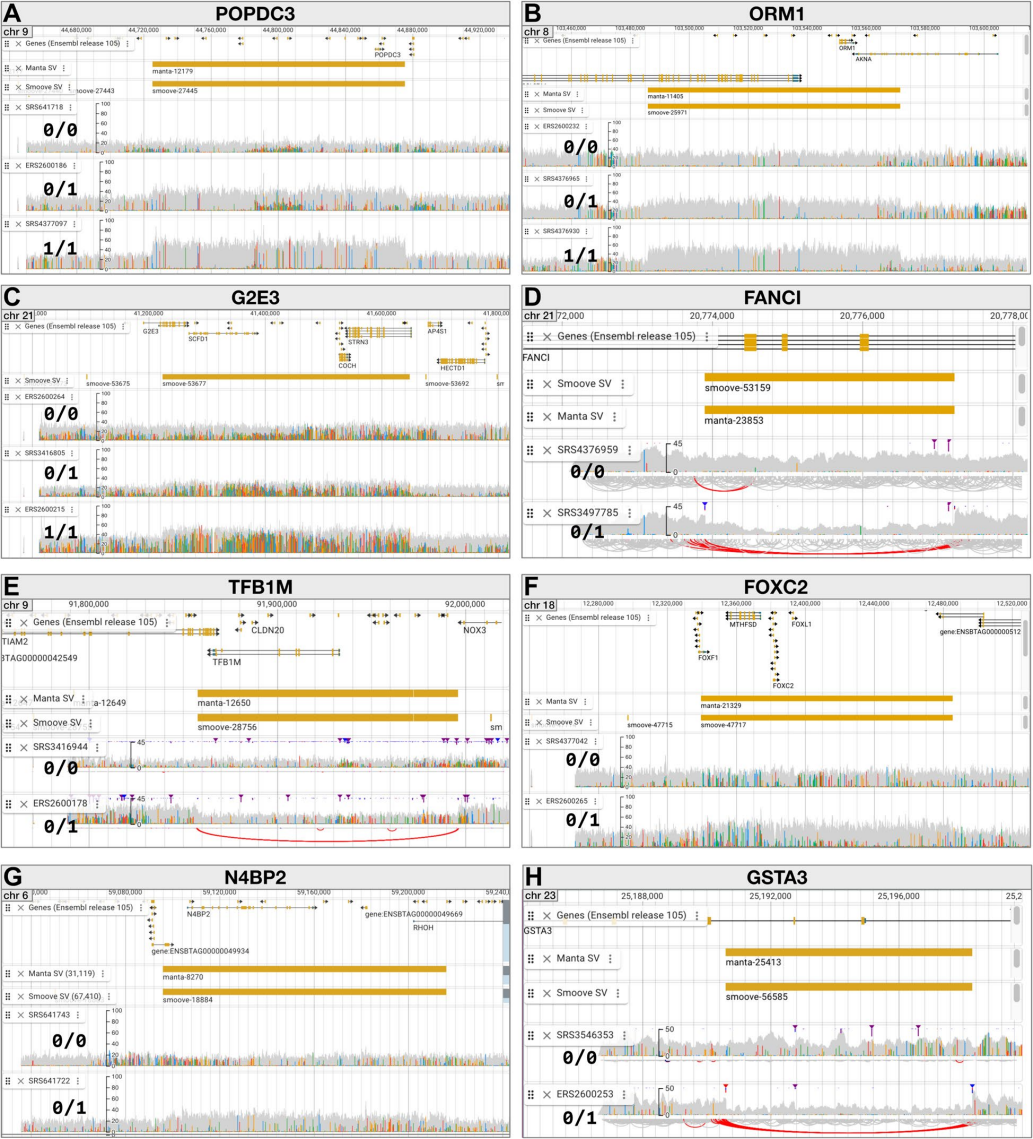
Examples of SVs visualized with the SVDB-DC genome viewer. Within each panel the top track displays genes from Ensembl (release 105) and is followed by tracks for the Manta and Smoove SVs (not necessarily in that order). The remaining tracks show read alignment (BAM) information for a single sample from each genotype category. The duplication examples (ABCFG) show alignment coverage while the deletion examples (DEH) show coverage and arcs representing long-range connections between reads

Using SVDB-DC, many examples of potentially gene-altering SVs can be identified and studied (Fig. 5). First, we searched SVDB-DC for several previously reported genic cattle duplications and deletions. Lee et al*.* [17] describe two predicted high-impact duplications: a 150 kb duplication overlapping the *POPDC3* gene and an 86 kb duplication overlapping the *ORM1* gene. Both are found in our dataset: searching for *POPDC3* in the SVDB-DC SVs panel returns the 150 kb duplication as found by both callers and classified as “shared” (manta-12179 and smoove-27445) (Fig. 5A); similarly, searching for *ORM1* returns the 86 kb duplication as identified by both Manta and Smoove (manta-11405 and smoove-25971) (Fig. 5B). This duplication was not classified as shared between callers due to the genotypes not reaching the required concordance threshold. Surprisingly, the *ORM1* duplications are not classified as high impact by VEP despite their extensive overlap with several genes. For this reason, we recommend using the included gene overlap information in SVDB-DC and Additional files 3 and 4 when prioritizing SVs.

Oliveira et al*.* [60] reported four copy number variant regions (CNVRs) associated with reproduction or disease traits, two of which exceed the 0.5 Mb size threshold for our database. The remaining two are a 36 kb duplication on BTA5 (CNVR1) that overlaps the *CD163L1* gene and a 43 kb duplication on BTA21 that overlaps the *G2E3* gene (CNVR3). CNVR3 is readily found in SVDB-DC as smoove-53677 (Fig. 5C). Multiple overlapping SVs are found in SVDB-DC for the *CD163L1* gene region, including large duplications reported by Manta (manta-7225, 83 kb) and Smoove (smoove-16775, 220 kb; smoove-16782, 81 kb). Due to the complexity of this region, it is difficult to match a particular SVDB-DC SV to CNVR1, but the results support that this region displays copy number variation.

Charlier et al*.* [9] identified a 3.3 kb deletion in the *FANCI* gene that leads to brachyspina syndrome, a rare recessive genetic defect in Holstein cattle. This SV is found in SVDB-DC as a shared site (manta-23853 and smoove-53159) (Fig. 5D). As with some other genic SVs in this dataset, VEP assigned a predicted impact rating of “low” despite the extensive gene overlap.

Schütz et al*.* [10] reported that lethal haplotype 5 [61] in Holstein cattle is a 138 kb deletion on chromosome 9 which encompasses the *TFB1M* gene. SVDB-DC contains this deletion as a shared site (manta-12650 and smoove-28756) (Fig. 5E).

For each of these previously described regions SVDB-DC provides a variety of useful information including precise SV boundaries across samples, allele and genotype frequency information, overlapping genes, overlapping SNPs, and tag SNPs.

### Examples of additional well-supported genic SVs in SVDB-DC

We looked for additional examples of well-supported (by visualization in SVDB-DC) SVs that overlap with known or predicted genes. A 146 kb duplication identified by both Manta and Smoove (manta-21329 and smoove-47717) encompass several genes including *FOXC2* (Fig. 5F). *FOXC2* is highly expressed in horn tissue and bone and may be involved in horn development [62]. A 120 kb duplication (manta-8270 and smoove-18884) contains the *N4BP2* gene (Fig. 5G). Variants of *N4BP2* may be associated with nonsyndromic cleft lip in humans [63]. An 8 kb deletion (manta-25413 and smoove-56585) includes a portion of the *GSTA3* gene (Fig. 5H), which is a member of the glutathione-S-transferases family of proteins best known for their roles as detoxification enzymes [64]. Interestingly, like the *FANCI* and *TFB1M* deletions, the *GSTA3* deletion is observed only in heterozygous form in this study population, raising the possibility that it too is harmful in homozygous form. However, given the deletion frequency in this sample set (0.06), the absence of the homozygous genotype could also be due to chance (HWE *p*-value 0.61).

#### Using SVDB-DC to determine SV boundaries

**Fig. 6:**
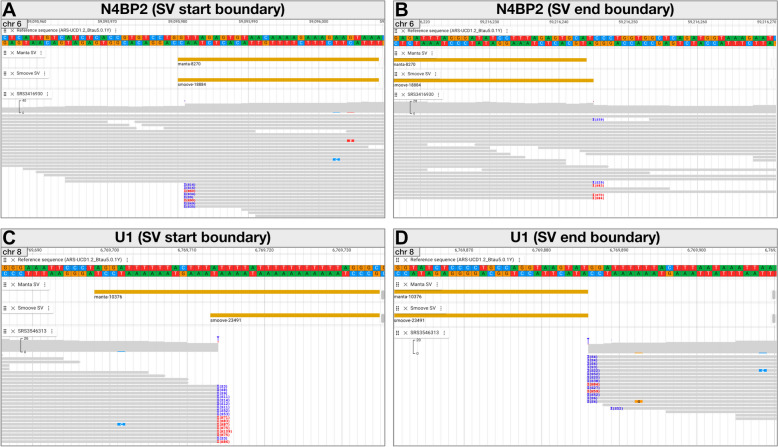
Precise structural variant boundary visualization with SVDB-DC. **A** Start and (**B**) end boundaries of SVs (manta-8270 and smoove-18884) overlapping the *N4BP2* gene. **C** Start and (**D**) end boundaries of SVs (manta-10376 and smoove-23491) overlapping the *U1* gene. Within each panel the top track displays the reference genome sequence (ARS-UCD1.2_Btau5.0.1Y) and is followed by tracks showing Manta and Smoove SVs. The bottom track shows coverage and a pileup display of the aligned reads with indicators for soft (blue) and hard (red) clipping

Although the locations of SVs can be parsed from a VCF file or another textual representation, it can be difficult to gauge the support of position information across samples and between SV callers. SVDB-DC permits visualization of VCF-extracted position information as SV features, which can then be compared to read alignments viewed in BAM tracks. Figure 6AB shows a comparison of the Manta and Smoove results (manta-8270 and smoove-18884) for the *N4BP2* duplication. Both representations start at the same position, but the end position differs by a single bp. Visualization of the read alignments supports the Smoove end position. Figure 6CD shows Manta and Smoove deletions (manta-10376 and smoove-23491) that overlap with a spliceosomal RNA gene; in this case zooming in on the SV boundaries shows that the end positions match and are supported by the read alignments, whereas the start positions differ, with the Smoove start position being the correct one. The SVs (manta-8270, smoove-18884, and smoove-23491) appear to begin 1 bp before the positions suggested by the read alignments (Fig. 6AC), however, this is a consequence of how these SVs are stored in VCFs. According to the VCF specification [65], for insertions and deletions where one of the REF or ALT alleles would be empty, or duplications with a symbolic allele (e.g. < DUP >), the start position includes the base before the event. This is the case for all the Manta and Smoove results.

#### Examining sex chromosome SVs

There were few Y chromosome SVs identified in the dataset (12 by Manta and 99 by Smoove). None overlap with exons based on the feature overlap analysis, and all were annotated as intergenic by VEP. On the X chromosome there were 1075 and 1761 SVs identified by Manta and Smoove, respectively. As with the Y chromosome, the density of SVs detected is low relative to the autosomes (Additional file 2). Among the X chromosome sites we did not find any strong candidate functionally important SVs (looking for SVs identified by both callers and that overlap with CDS exons).

The ability to group samples by sex in SVDB-DC is useful for interpreting sex chromosome SVs. Additional file 5 shows the manta-30188 / smoove-65778 deletion, which is located within an intron of the *ATP1B4* (ATPase Na + /K + transporting family member beta 4) gene. Grouping samples by sex using the SVDB-DC interface convincingly shows the presence of a heterozygous female and three hemizygous males. Without sex information it is more difficult to assess the read coverage results and genotype frequencies: if the samples are all interpreted as male, then the heterozygous sample would appear to be incorrectly genotyped, and if the samples are treated as female, then the genotypes appear to be suspect due to the departure from HWE expectations (*p* = 4.47 × 10⁻⁷).

#### Deletion artifacts from processed pseudogenes

**Fig. 7:**
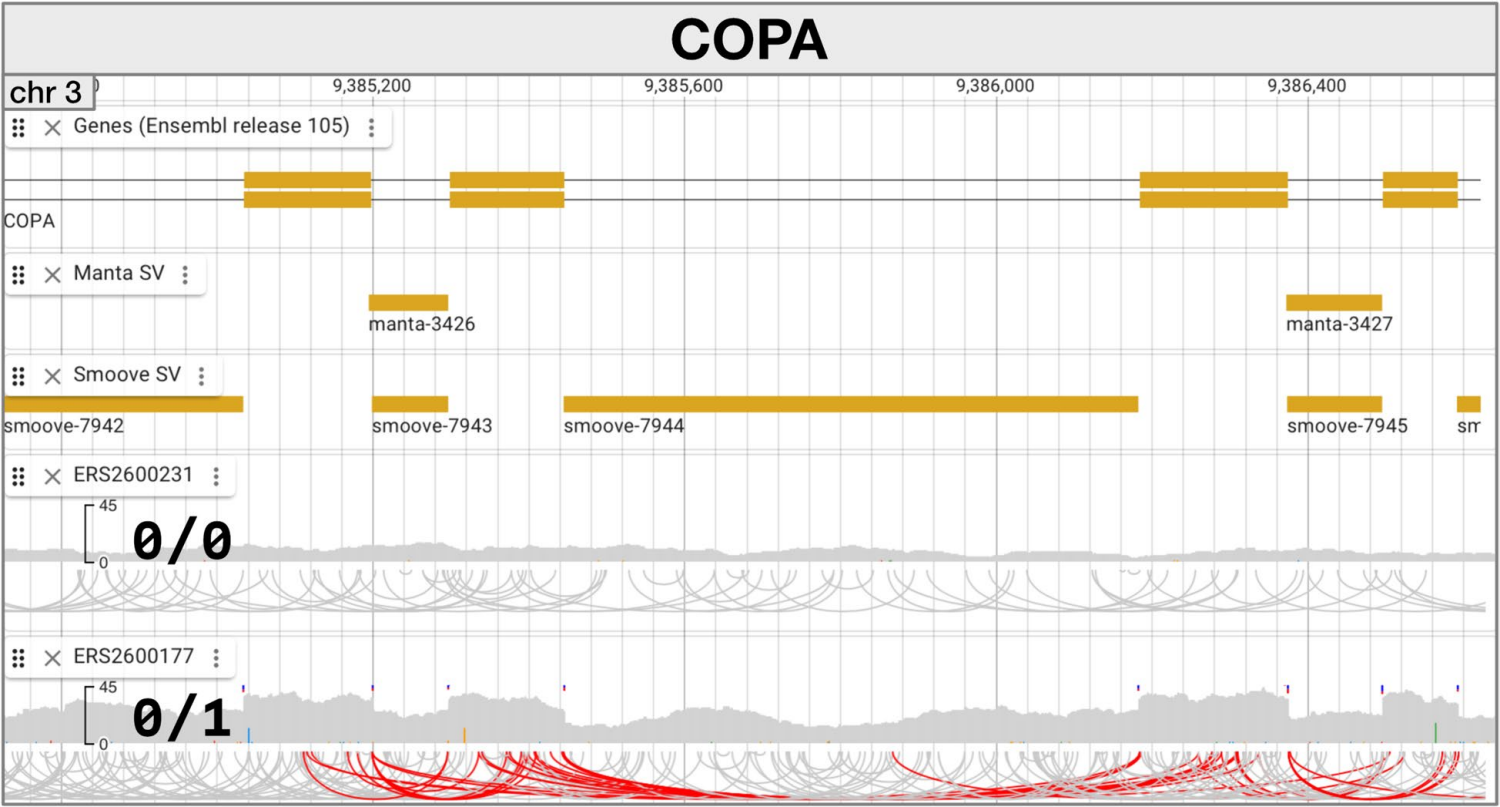
Artifactual deletions in the *COPA* gene caused by a processed pseudogene. A portion of two Ensembl *COPA* gene models is shown (top track), followed by Manta and Smoove SVs, and then read alignment data for an individual lacking the deletions (0/0) and the single individual (ERS2600177) with the deletions. Exons in the *COPA* gene (rectangles) align with higher coverage regions in ERS2600177 and the Manta and Smoove deletions align with introns in *COPA*

Visualizing read alignments in SVDB-DC in gene regions can reveal cases where deletions correspond exactly to or close to intronic segments. Often such deletions affect multiple adjacent introns or sometimes all the introns in a gene. Figure 7 shows an example involving the *COPA* gene. A single individual in this study (ERS2600177) has a series of apparent intronic deletions in *COPA*. Meanwhile, the *COPA* exons in this sample show unusually high coverage in comparison to other samples. Both characteristics are consistent with reads from a *COPA* processed pseudogene locus aligning to the reference gene. Both Manta and Smoove are making reasonable predictions in the case of these artifactual deletions: the reads from the processed pseudogene align with the reference gene, and those that span exon boundaries are split just like those produced from genuine deletions. Further inspection of the read alignments in this individual revealed that the processed pseudogene-derived reads come from an integration event on chromosome 7 (not shown). Visual assessment of sites with notable VEP impacts or feature overlaps using SVDB-DC is recommended given the potential of processed pseudogenes to lead to deletion SV calls in gene regions. The boundaries of these SVs sometimes overlap with or span exons, leading to exon overlaps being reported, and in many cases moderate or high-impact predictions. Signs we take to suggest involvement of a processed pseudogene include deletions with boundaries close to or matching intron boundaries, higher coverage of the adjacent exon regions than background levels (due to the reads from the processed pseudogene aligning with the reference gene), a succession of multiple deletion calls from the same individual(s) affecting a series of introns in the same gene, and predominantly or exclusively heterozygous deletion genotypes.

## Discussion

In the present study we aimed to provide an updated, thoroughly annotated and characterized set of cattle SVs from two SV callers. Furthermore, we sought to present the results in a way that allows us and others to make full use of the results in ongoing efforts to link specific DNA differences to phenotypic variation. Although we conducted a large-scale SV analysis in cattle previously [21], the current effort supersedes this previous work in terms of the quality of input data, the performance of the SV callers, the quality of the reference assembly (UMD 3.1 in the previous study and ARS-UCD1.2 with the Y chromosome from assembly Btau5.0.1 in this work), the comprehensiveness of the SV characterization and annotation approaches, the handling of sample sex and sex chromosomes, and the approach used for data sharing and visualization. Most notably, the SVDB-DC allows the underlying supporting read data for any SV in the dataset to be scrutinized down to the nucleotide level, and provides access to the raw genotypes, annotations, and other analysis results (e.g. tag SNPs) generated.

The characteristics of the Holstein cattle SVs we identified support the potential importance of SVs in contributing to phenotypic variation in this breed: they cover a greater portion of the reference genome in the study population than SNPs and indels; most are polymorphic within the group of animals studied; and variant effect prediction and feature overlaps suggest potential moderate or high impacts on a variety of genes. With the caveat that SV calls from short-read are of modest accuracy [66–68] and that functional predictions are limited by incomplete knowledge of the roles of reference genome regions and by the complexity of biological systems, the results support continued efforts to identify and understand the roles of SVs in this breed.

To reduce the presence of false positive SVs, a variety of strategies have been applied in previous work, for example requiring that SVs be identified by multiple approaches [17, 29, 31], and filtering based on population frequency [69]. A limitation of the present work is the permissive filtering used to generate the SVs, which we would expect to lead to more false-positive SVs in our dataset compared to those generated using more stringent filters. With the goal of building a large catalogue of putative SVs that can each be further examined visually, we did not apply downstream filtering of SV results beyond what was recommended by the Manta and Smoove developers. Using the SV database refined sets of SVs can be generated through filtering on frequency, calling program, HWE p-value, and a variety of other criteria. The SV VCF files are also available from SVDB-DC, allowing others to apply custom filtering approaches programmatically.

There are several other studies that have identified SVs in large populations of cattle using Smoove. In Bhati et al*.* [70], Smoove identified 61,806 SVs in 183 cattle genomes, similar to the 67,550 that we found. Lee et al. [17] identified 13,731 SVs in 266 Holstein–Friesian samples (prior to filtering) with Smoove. Another popular tool for SV detection is CNVnator [71], which both we and others have used. In a previous study using CNVnator, we found 124,302 CNV regions in 96 Holstein cattle [29] whereas Couldrey et al. [26] found 43,708 CNVs with the same tool in 556 Holstein-Friesians, Jerseys, and Holstein–Friesian x Jersey crossbreeds. Differences in population size and makeup, library generation and sequencing methods, SV calling software and parameter use, and data filtering are all potential reasons for the large variation in the numbers and sizes of structural variants detected in these studies and make comparisons between datasets challenging.

Although we did not filter the SV results, we applied a quality control step in the form of comparing kinship coefficients calculated using the SV genotypes from Manta and Smoove to those calculated using SNPs. This step is valuable in that it can reveal problems in data handling; for example, if genotypes from any of the callers are matched incorrectly to samples (e.g. through assuming samples are ordered in a particular way across VCF files) the correlation can be lost altogether. In the absence of trio information or a set of independently established “gold standard” SVs, the examination of kinship correlations also gave us a means to compare genotype quality among the different SV types and between Manta and Smoove within the study dataset. Our findings that deletions performed better than duplications is consistent with formal evaluations of SV callers showing that deletions are more accurately called by SV callers than duplications [66, 68]. Generally, higher coverage data results in more precise SV calls [72], particularly for allelic copy number variations [16]. Downsampling the raw read datasets to improve computational resource use may have impacted SV detection and genotyping, especially in regions of lower-than-average coverage. For all variant types, Manta genotypes produced higher kinship coefficient correlations with SNPs than did Smoove genotypes. These results reflect the filtering steps applied and could change if further filtering were performed. A recent comparison of SV callers using high-quality sets of known variants and that included Manta and Smoove (Smoove is used to run Lumpy) reported Manta as the top performer of all the programs [67]. As far as the practical implications of these findings on the use of SVDB-DC we suggest using the Manta genotypes for the sites in our study that were identified by both callers and treating duplications with more skepticism than deletions. However, for determining the end point of such SVs we find that Smoove often gives more accurate results (see examples in Fig. 5).

An important part of identifying well-supported SV genotypes and of refining SV boundaries is the visualization of read alignments [73, 74]. Historically a major challenge in this regard is the user interaction and time required for loading the requisite BAM files into a visualization tool like IGV [75] and navigating to regions of interest. To circumvent this issue the SV-plaudit framework can be used to create a gallery of static read alignment and SV signal images for manual assessment [74]. However, SV-plaudit images don’t support zooming, customization of the display, or accessing the underlying BAM file fields to explore specific aspects of the raw data. For this reason, we built a custom web-based database (SVDB-DC) that uses JBrowse 2 to display BAM file information from a selection of samples chosen to represent the different genotype classes observed for the SV. We also created a searchable list of SVs and a URL API that can be used to quickly navigate to SVs of interest. It is important to note that no matter how convenient the visualization system, it is not feasible for a person to inspect all the SVs. Instead, the intention is to allow loci or SVs of interest to be studied in detail.

The utility of the integrated read alignments was shown for several SVs, providing support for SV genotypes and allowing SV positions to be refined. In the absence of functional evidence linking sequencing-discovered SVs to phenotype we can only speculate about the potential phenotypic relevance of the sequence variation. However, the tag and overlapping SNPs provided in SVDB-DC may prove useful in their study. Although we focus on genic SVs, those that do not overlap with genes may be of interest as well. Indeed, the 80-kb DNA duplication on BTA1 that is associated with horn absence [7] can be readily found in our dataset, having been identified by both Manta (manta-37) and Smoove (smoove-80) (data not shown).

Inspecting read alignments in SVDB-DC revealed that processed pseudogenes likely produce some of the gene region deletions in the dataset, and an example involving the *COPA* gene was featured. Processed pseudogenes are known to be variable across populations, and recent studies have focused on their identification using SV calling tools or other approaches [76, 77]. For loci that are genotyped for breeding or management purposes, which is true of *COPA* since it affects coat colour [78], the presence of a processed pseudogene can be problematic if it leads to incorrect or missing genotypes [79], through the unintentional assessment of the processed pseudogene sequence. The *COPA* processed pseudogene reported here appears to have a mutation (relative to the source locus) immediately adjacent to the Dominant Red (DR) mutation site, but the DR site (in the pseudogene) matches that of the ancestral wild-type allele, suggesting that it won’t lead to incorrect DR genotypes. Some processed pseudogenes are biologically relevant: even though they tend to accumulate mutations preventing the production of a functional protein, they can be transcribed to produce antisense RNA that interferes with the activity of the parental gene and some contain ORFs that produce biologically relevant proteins (reviewed in Troskie et al. [80]).

Confirming functional roles of SVs from this study and others requires genetic or molecular studies. The former have been done in cattle [60, 81] using SNP chip genotypes and signal intensity measures that are converted to CNV genotypes using programs like PennCNV [54] and QuantiSNP [55]. Although these approaches have successfully identified associations with very large SVs [60, 81], our findings suggest that most of the SVs in Holstein do not overlap with SNPs on the BovineSNP50 or BovineHD SNP chips, and thus can’t be assessed using this approach. Linkage disequilibrium between SNPs and an SV locus can permit the effects of the SV to be identified using tag SNPs. Based on our results most of the SVs in this dataset are not tagged by a SNP on the BovineSNP50 or BovineHD SNP chips, again limiting the utility of these chips for studying these SVs. Nearly half of the Manta SVs and 23% of the Smoove SVs have a WGS tag SNP, raising the possibility of adding new tag markers to SNP chips for SVs of interest. As for molecular studies of function, the SVDB-DC with its ability to display SV boundaries across individuals in conjunction with genome feature information could be useful in developing research strategies and reagents (e.g. molecular probes or primers for genotyping, measuring gene expression, or modifying gene activity).

An alternative to direct genotyping of individuals for SVs is to use imputation to infer SV genotypes in individuals with SNP chip genotypes [31, 82]. Long-read sequencing is especially exciting in this regard since the reads can capture the entirety of many SV alleles and can more often be unambiguously aligned to the reference genome, which should lead to a more accurate and comprehensive catalogue of SV locations and genotypes [5]. Even with the anticipated migration to better sequencing technologies the results of the present study will remain useful as a source of SNP, indel, and SV genotypes and annotations. Moreover, the data visualization and sharing approach developed here can be adapted to future datasets to increase the utility of the data.

In summary, this large collection of SV, SNP, and indel genotypes from 310 Holstein cattle, the accompanying database that supports the inspection of SV regions to the nucleotide level, and the associated functional predictions, tag, and overlapping SNP information can be used in ongoing efforts to study SVs and to use SV information in the improvement of this important breed.

## Supplementary Information

Additional file 1. Samples used in this study. Excel file of sample information including NCBI BioProject, BioSample, and SRA sample identifiers, sequence coverage metrics, sex as reported in the BioSample metadata, and inferred sex from sequence coverage.

Additional file 2. Distribution of Manta and Smoove SVs and genes in the cattle genome. Each chromosome is represented by three tracks: Manta (top), Smoove (middle) and genes (bottom). The Manta and Smoove tracks show duplications and deletions as orange rectangles and the gene track shows genes as blue rectangles. All tracks are overlaid with density plots of the features in the track using a window size of 500 kb (black). The X-axis is the chromosome position in Mb. The plot was created with the R package karyoploteR.

Additional file 3. Manta structural variants. Excel file of Manta SVs, including genotype summary information, VEP impacts, overlapping genes, overlapping panel SNPs, tag SNPs, and links to visualize read alignments.

Additional file 4. Smoove structural variants. Excel file of Smoove SVs, including genotype summary information, VEP impacts, overlapping genes, overlapping panel SNPs, tag SNPs, and links to visualize read alignments.

Additional file 5. Sex chromosome SV visualized with SVDB-DC. The manta-30188 / smoove-65778 deletion, located on chromosome X, is shown in (A) female and (B) male samples. The selected sex (red box) restricts the genotype counts (orange box) and the BAM tracks to that sex. The “Genes (NCBI release 106)” track displays gene feature information from NCBI. The two horizontal lines in this track extending the width of the view represent an intron shared by two isoforms (or transcript variants) of the *ATP1B4* (ATPase Na+/K+ transporting family member beta 4) gene. The remaining tracks show the Manta and Smoove SVs followed by the read alignment (BAM) tracks for a single sample from each genotype category.

## Data Availability

SV data is provided within the supplementary information files. SVDB-DC is freely accessible from https://svdb-dc.pslab.ca.

## References

[1] Daetwyler HD, Capitan A, Pausch H, Stothard P, van Binsbergen R, Brøndum RF, et al. Whole-genome sequencing of 234 bulls facilitates mapping of monogenic and complex traits in cattle. Nat Genet. 2014;46(8):858–65. 25017103 10.1038/ng.3034

[2] Dutta P, Talenti A, Young R, Jayaraman S, Callaby R, Jadhav SK, et al. Whole genome analysis of water buffalo and global cattle breeds highlights convergent signatures of domestication. Nat Commun. 2020;11(1):4739. 32958756 10.1038/s41467-020-18550-1 PMC7505982

[3] Li X, Yang J, Shen M, Xie XL, Liu GJ, Xu YX, et al. Whole-genome resequencing of wild and domestic sheep identifies genes associated with morphological and agronomic traits. Nat Commun. 2020;11(1):2815. 32499537 10.1038/s41467-020-16485-1 PMC7272655

[4] Tan X, Liu L, Liu X, Cui H, Liu R, Zhao G, et al. Large-Scale Whole Genome Sequencing Study Reveals Genetic Architecture and Key Variants for Breast Muscle Weight in Native Chickens. Genes. 2022;13(1):3. 10.3390/genes13010003 PMC8774586 35052342

[5] Nguyen TV, Vander Jagt CJ, Wang J, Daetwyler HD, Xiang R, Goddard ME, et al. In it for the long run: perspectives on exploiting long-read sequencing in livestock for population scale studies of structural variants. Genet Sel Evol GSE. 2023;55(1):9. 36721111 10.1186/s12711-023-00783-5 PMC9887926

[6] Medugorac I, Seichter D, Graf A, Russ I, Blum H, Göpel KH, et al. Bovine polledness–an autosomal dominant trait with allelic heterogeneity. PLoS ONE. 2012;7(6): e39477. 22737241 10.1371/journal.pone.0039477 PMC3380827

[7] Rothammer S, Capitan A, Mullaart E, Seichter D, Russ I, Medugorac I. The 80-kb DNA duplication on BTA1 is the only remaining candidate mutation for the polled phenotype of Friesian origin. Genet Sel Evol. 2014;46(1):44. 24993890 10.1186/1297-9686-46-44 PMC4099407

[8] Durkin K, Coppieters W, Drögemüller C, Ahariz N, Cambisano N, Druet T, et al. Serial translocation by means of circular intermediates underlies colour sidedness in cattle. Nature. 2012;482(7383):81–4. 22297974 10.1038/nature10757

[9] Charlier C, Agerholm JS, Coppieters W, Karlskov-Mortensen P, Li W, de Jong G, et al. A deletion in the bovine FANCI gene compromises fertility by causing fetal death and brachyspina. PLoS ONE. 2012;7(8): e43085. 22952632 10.1371/journal.pone.0043085 PMC3430679

[10] Schütz E, Wehrhahn C, Wanjek M, Bortfeld R, Wemheuer WE, Beck J, et al. The Holstein Friesian Lethal Haplotype 5 (HH5) Results from a Complete Deletion of TBF1M and Cholesterol Deficiency (CDH) from an ERV-(LTR) Insertion into the Coding Region of APOB. PLoS ONE. 2016;11(4): e0154602. 27128314 10.1371/journal.pone.0154602 PMC4851415

[11] Mei C, Junjvlieke Z, Raza SHA, Wang H, Cheng G, Zhao C, et al. Copy number variation detection in Chinese indigenous cattle by whole genome sequencing. Genomics. 2020;112(1):831–6. 31145994 10.1016/j.ygeno.2019.05.023

[12] Xia X, Zhang F, Li S, Luo X, Peng L, Dong Z, et al. Structural variation and introgression from wild populations in East Asian cattle genomes confer adaptation to local environment. Genome Biol. 2023;24(1):211. 37723525 10.1186/s13059-023-03052-2 PMC10507960

[13] Liu GE, Brown T, Hebert DA, Cardone MF, Hou Y, Choudhary RK, et al. Initial analysis of copy number variations in cattle selected for resistance or susceptibility to intestinal nematodes. Mamm Genome Off J Int Mamm Genome Soc. 2011;22(1–2):111–21. 10.1007/s00335-010-9308-0 21125402

[14] Hou Y, Liu GE, Bickhart DM, Matukumalli LK, Li C, Song J, et al. Genomic regions showing copy number variations associate with resistance or susceptibility to gastrointestinal nematodes in Angus cattle. Funct Integr Genomics. 2012;12(1):81–92. 21928070 10.1007/s10142-011-0252-1

[15] Xu L, Hou Y, Bickhart DM, Song J, Van Tassell CP, Sonstegard TS, et al. A genome-wide survey reveals a deletion polymorphism associated with resistance to gastrointestinal nematodes in Angus cattle. Funct Integr Genomics. 2014;14(2):333–9. 24718732 10.1007/s10142-014-0371-6

[16] Lee YL, Takeda H, Costa Monteiro Moreira G, Karim L, Mullaart E, Coppieters W, et al. A 12 kb multi-allelic copy number variation encompassing a GC gene enhancer is associated with mastitis resistance in dairy cattle. PLoS Genet. 2021;17(7):e1009331. 34288907 10.1371/journal.pgen.1009331 PMC8328317

[17] Lee YL, Bosse M, Takeda H, Moreira GCM, Karim L, Druet T, et al. High-resolution structural variants catalogue in a large-scale whole genome sequenced bovine family cohort data. BMC Genomics. 2023;24(1):225. 37127590 10.1186/s12864-023-09259-8 PMC10152703

[18] Hou Y, Bickhart DM, Chung H, Hutchison JL, Norman HD, Connor EE, et al. Analysis of copy number variations in Holstein cows identify potential mechanisms contributing to differences in residual feed intake. Funct Integr Genomics. 2012;12(4):717–23. 22991089 10.1007/s10142-012-0295-y

[19] McDaneld TG, Kuehn LA, Thomas MG, Pollak EJ, Keele JW. Deletion on chromosome 5 associated with decreased reproductive efficiency in female cattle. J Anim Sci. 2014;92(4):1378–84. 24492568 10.2527/jas.2013-6821

[20] Kadri NK, Sahana G, Charlier C, Iso-Touru T, Guldbrandtsen B, Karim L, et al. A 660-Kb deletion with antagonistic effects on fertility and milk production segregates at high frequency in Nordic Red cattle: additional evidence for the common occurrence of balancing selection in livestock. PLoS Genet. 2014;10(1): e1004049. 24391517 10.1371/journal.pgen.1004049 PMC3879169

[21] Kommadath A, Grant JR, Krivushin K, Butty AM, Baes CF, Carthy TR, et al. A large interactive visual database of copy number variants discovered in taurine cattle. GigaScience. 2019;8(6):giz073. 31241156 10.1093/gigascience/giz073 PMC6593363

[22] Zhou Y, Yang L, Han X, Han J, Hu Y, Li F, et al. Assembly of a pangenome for global cattle reveals missing sequences and novel structural variations, providing new insights into their diversity and evolutionary history. Genome Res. 2022;32(8):1585–601. 35977842 10.1101/gr.276550.122 PMC9435747

[23] Mesbah-Uddin M, Guldbrandtsen B, Iso-Touru T, Vilkki J, De Koning DJ, Boichard D, et al. Genome-wide mapping of large deletions and their population-genetic properties in dairy cattle. DNA Res Int J Rapid Publ Rep Genes Genomes. 2018;25(1):49–59. 10.1093/dnares/dsx037 PMC5824824 28985340

[24] Chen L, Chamberlain AJ, Reich CM, Daetwyler HD, Hayes BJ. Detection and validation of structural variations in bovine whole-genome sequence data. Genet Sel Evol. 2017;49(1):13. 28122487 10.1186/s12711-017-0286-5 PMC5267451

[25] Boussaha M, Esquerré D, Barbieri J, Djari A, Pinton A, Letaief R, et al. Genome-Wide Study of Structural Variants in Bovine Holstein, Montbéliarde and Normande Dairy Breeds. PLoS ONE. 2015;10(8): e0135931. 26317361 10.1371/journal.pone.0135931 PMC4552564

[26] Couldrey C, Keehan M, Johnson T, Tiplady K, Winkelman A, Littlejohn MD, et al. Detection and assessment of copy number variation using PacBio long-read and Illumina sequencing in New Zealand dairy cattle. J Dairy Sci. 2017;100(7):5472–8. 28456410 10.3168/jds.2016-12199

[27] Mielczarek M, Frąszczak M, Giannico R, Minozzi G, Williams JL, Wojdak-Maksymiec K, et al. Analysis of copy number variations in Holstein-Friesian cow genomes based on whole-genome sequence data. J Dairy Sci. 2017;100(7):5515–25. 28501396 10.3168/jds.2016-11987

[28] Hu Y, Xia H, Li M, Xu C, Ye X, Su R, et al. Comparative analyses of copy number variations between Bos taurus and Bos indicus. BMC Genomics. 2020;21(1):682. 33004001 10.1186/s12864-020-07097-6 PMC7528262

[29] Butty AM, Chud TCS, Miglior F, Schenkel FS, Kommadath A, Krivushin K, et al. High confidence copy number variants identified in Holstein dairy cattle from whole genome sequence and genotype array data. Sci Rep. 2020;10(1):8044. 32415111 10.1038/s41598-020-64680-3 PMC7229195

[30] Upadhyay M, Derks MFL, Andersson G, Medugorac I, Groenen MAM, Crooijmans RPMA. Introgression contributes to distribution of structural variations in cattle. Genomics. 2021;113(5):3092–102. 34242710 10.1016/j.ygeno.2021.07.005

[31] Chen L, Pryce JE, Hayes BJ, Daetwyler HD. Investigating the Effect of Imputed Structural Variants from Whole-Genome Sequence on Genome-Wide Association and Genomic Prediction in Dairy Cattle. Anim Open Access J MDPI. 2021;11(2):541. 10.3390/ani11020541 PMC7922624 33669735

[32] Pedersen BS, Quinlan AR. Mosdepth: quick coverage calculation for genomes and exomes. Bioinforma Oxf Engl. 2018;34(5):867–8. 10.1093/bioinformatics/btx699 PMC6030888 29096012

[33] Bolger AM, Lohse M, Usadel B. Trimmomatic: a flexible trimmer for Illumina sequence data. Bioinforma Oxf Engl. 2014;30(15):2114–20. 10.1093/bioinformatics/btu170 PMC4103590 24695404

[34] Rosen BD, Bickhart DM, Schnabel RD, Koren S, Elsik CG, Tseng E, et al. De novo assembly of the cattle reference genome with single-molecule sequencing. GigaScience. 2020;9(3):giaa021. 32191811 10.1093/gigascience/giaa021 PMC7081964

[35] Li H, Durbin R. Fast and accurate short read alignment with Burrows-Wheeler transform. Bioinforma Oxf Engl. 2009;25(14):1754–60. 10.1093/bioinformatics/btp324 PMC2705234 19451168

[36] DePristo MA, Banks E, Poplin R, Garimella KV, Maguire JR, Hartl C, et al. A framework for variation discovery and genotyping using next-generation DNA sequencing data. Nat Genet. 2011;43(5):491–8. 21478889 10.1038/ng.806 PMC3083463

[37] Chen X, Schulz-Trieglaff O, Shaw R, Barnes B, Schlesinger F, Källberg M, et al. Manta: rapid detection of structural variants and indels for germline and cancer sequencing applications. Bioinforma Oxf Engl. 2016;32(8):1220–2. 10.1093/bioinformatics/btv710 26647377

[38] Pedersen BS, Layer R, Quinlan AR. smoove: structural-variant calling and genotyping with existing tools [Internet]. 2020 [cited 2024 Apr 22]. Available from: https://github.com/brentp/smoove.

[39] Danecek P, Bonfield JK, Liddle J, Marshall J, Ohan V, Pollard MO, et al. Twelve years of SAMtools and BCFtools. GigaScience. 2021;10(2):giab008. 33590861 10.1093/gigascience/giab008 PMC7931819

[40] Danecek P, Auton A, Abecasis G, Albers CA, Banks E, DePristo MA, et al. The variant call format and VCFtools. Bioinformatics. 2011;27(15):2156–8. 21653522 10.1093/bioinformatics/btr330 PMC3137218

[41] pysam-developers/pysam [Internet]. pysam-developers; 2024 [cited 2024 Apr 22]. Available from: https://github.com/pysam-developers/pysam.

[42] Li H, Handsaker B, Wysoker A, Fennell T, Ruan J, Homer N, et al. The Sequence Alignment/Map format and SAMtools. Bioinforma Oxf Engl. 2009;25(16):2078–9. 10.1093/bioinformatics/btp352 PMC2723002 19505943

[43] Gel B, Serra E. karyoploteR: an R/Bioconductor package to plot customizable genomes displaying arbitrary data. Bioinformatics. 2017;33(19):3088–90. 28575171 10.1093/bioinformatics/btx346 PMC5870550

[44] Stothard P. paulstothard/identify-shared-SVs [Internet]. 2024 [cited 2024 Sep 10]. Available from: https://github.com/paulstothard/identify-shared-SVs.

[45] Manichaikul A, Mychaleckyj JC, Rich SS, Daly K, Sale M, Chen WM. Robust relationship inference in genome-wide association studies. Bioinforma Oxf Engl. 2010;26(22):2867–73. 10.1093/bioinformatics/btq559 PMC3025716 20926424

[46] Stothard P. paulstothard/genotype_conversion_file_builder [Internet]. 2024 [cited 2024 May 8]. Available from: https://github.com/paulstothard/genotype_conversion_file_builder.

[47] McKinney W. Data structures for statistical computing in Python. In: Van der Walt S, Millman J, editors. Proceedings of the 9th Python in Science Conference. Austin (TX): SciPy; 2010. p. 56–61. 10.25080/Majora-92bf1922-00a.

[48] The pandas development team. pandas-dev/pandas: Pandas [Internet]. Zenodo; 2020. Available from: 10.5281/zenodo.8364959.

[49] Cezard T, Cunningham F, Hunt SE, Koylass B, Kumar N, Saunders G, et al. The European Variation Archive: a FAIR resource of genomic variation for all species. Nucleic Acids Res. 2022;50(D1):D1216–20. 34718739 10.1093/nar/gkab960 PMC8728205

[50] Quinlan AR, Hall IM. BEDTools: a flexible suite of utilities for comparing genomic features. Bioinforma Oxf Engl. 2010;26(6):841–2. 10.1093/bioinformatics/btq033 PMC2832824 20110278

[51] Digital Research Alliance of Canada [Internet]. 2024 [cited 2024 Apr 24]. Digital Research Alliance of Canada. Available from: https://alliancecan.ca/en/node/10.

[52] React [Internet]. [cited 2024 Apr 24]. Available from: https://react.dev/.

[53] Diesh C, Stevens GJ, Xie P, De Jesus MT, Hershberg EA, Leung A, et al. JBrowse 2: a modular genome browser with views of synteny and structural variation. Genome Biol. 2023;24(1):74. 37069644 10.1186/s13059-023-02914-z PMC10108523

[54] Wang K, Li M, Hadley D, Liu R, Glessner J, Grant SFA, et al. PennCNV: An integrated hidden Markov model designed for high-resolution copy number variation detection in whole-genome SNP genotyping data. Genome Res. 2007;17(11):1665–74. 17921354 10.1101/gr.6861907 PMC2045149

[55] Colella S, Yau C, Taylor JM, Mirza G, Butler H, Clouston P, et al. QuantiSNP: an Objective Bayes Hidden-Markov Model to detect and accurately map copy number variation using SNP genotyping data. Nucleic Acids Res. 2007;35(6):2013–25. 17341461 10.1093/nar/gkm076 PMC1874617

[56] McLaren W, Gil L, Hunt SE, Riat HS, Ritchie GRS, Thormann A, et al. The Ensembl Variant Effect Predictor. Genome Biol. 2016;17(1):122. 27268795 10.1186/s13059-016-0974-4 PMC4893825

[57] Rangwala SH, Kuznetsov A, Ananiev V, Asztalos A, Borodin E, Evgeniev V, et al. Accessing NCBI data using the NCBI Sequence Viewer and Genome Data Viewer (GDV). Genome Res. 2021;31(1):159–69. 33239395 10.1101/gr.266932.120 PMC7849379

[58] Nassar LR, Barber GP, Benet-Pagès A, Casper J, Clawson H, Diekhans M, et al. The UCSC Genome Browser database: 2023 update. Nucleic Acids Res. 2023;51(D1):D1188–95. 36420891 10.1093/nar/gkac1072 PMC9825520

[59] Martin FJ, Amode MR, Aneja A, Austine-Orimoloye O, Azov AG, Barnes I, et al. Ensembl 2023. Nucleic Acids Res. 2023;51(D1):D933–41. 36318249 10.1093/nar/gkac958 PMC9825606

[60] Oliveira HR, Chud TCS, Oliveira GA, Hermisdorff IC, Narayana SG, Rochus CM, et al. Genome-wide association analyses reveals copy number variant regions associated with reproduction and disease traits in Canadian Holstein cattle. J Dairy Sci. 2024;S0022–0302(24):00810–5. 10.3168/jds.2023-24295 38788846

[61] Fritz S, Capitan A, Djari A, Rodriguez SC, Barbat A, Baur A, et al. Detection of haplotypes associated with prenatal death in dairy cattle and identification of deleterious mutations in GART, SHBG and SLC37A2. PLoS ONE. 2013;8(6): e65550. 23762392 10.1371/journal.pone.0065550 PMC3676330

[62] Aldersey JE, Sonstegard TS, Williams JL, Bottema CDK. Understanding the effects of the bovine POLLED variants. Anim Genet. 2020;51(2):166–76. 31999853 10.1111/age.12915

[63] Leslie EJ, Carlson JC, Shaffer JR, Buxó CJ, Castilla EE, Christensen K, et al. Association studies of low-frequency coding variants in nonsyndromic cleft lip with or without cleft palate. Am J Med Genet A. 2017;173(6):1531–8. 28425186 10.1002/ajmg.a.38210 PMC5444956

[64] Mazari AMA, Zhang L, Ye ZW, Zhang J, Tew KD, Townsend DM. The Multifaceted Role of Glutathione S-Transferases in Health and Disease. Biomolecules. 2023;13(4):688. 37189435 10.3390/biom13040688 PMC10136111

[65] HTS format specifications [Internet]. [cited 2024 Jun 27]. Available from: https://samtools.github.io/hts-specs/.

[66] Kosugi S, Momozawa Y, Liu X, Terao C, Kubo M, Kamatani Y. Comprehensive evaluation of structural variation detection algorithms for whole genome sequencing. Genome Biol. 2019;20(1):117. 31159850 10.1186/s13059-019-1720-5 PMC6547561

[67] Sarwal V, Niehus S, Ayyala R, Kim M, Sarkar A, Chang S, et al. A comprehensive benchmarking of WGS-based deletion structural variant callers. Brief Bioinform. 2022;23(4):bbac221. 35753701 10.1093/bib/bbac221 PMC9294411

[68] Joe S, Park JL, Kim J, Kim S, Park JH, Yeo MK, et al. Comparison of structural variant callers for massive whole-genome sequence data. BMC Genomics. 2024;25(1):318. 38549092 10.1186/s12864-024-10239-9 PMC10976732

[69] Benfica LF, Brito LF, do Bem RD, de Oliveira LF, Mulim HA, Braga LG, et al. Detection and characterization of copy number variation in three differentially-selected Nellore cattle populations. Front Genet. 2024;15:1377130. 38694873 10.3389/fgene.2024.1377130 PMC11061390

[70] Bhati M, Mapel XM, Lloret-Villas A, Pausch H. Structural variants and short tandem repeats impact gene expression and splicing in bovine testis tissue. Genetics. 2023;225(3):iyad161. 37655920 10.1093/genetics/iyad161 PMC10627265

[71] Abyzov A, Urban AE, Snyder M, Gerstein M. CNVnator: An approach to discover, genotype, and characterize typical and atypical CNVs from family and population genome sequencing. Genome Res. 2011;21(6):974–84. 21324876 10.1101/gr.114876.110 PMC3106330

[72] Raca G, Astbury C, Behlmann A, De Castro MJ, Hickey SE, Karaca E, et al. Points to consider in the detection of germline structural variants using next-generation sequencing: A statement of the American College of Medical Genetics and Genomics (ACMG). Genet Med Off J Am Coll Med Genet. 2023;25(2): 100316. 10.1016/j.gim.2022.09.017 36507974

[73] Trost B, Walker S, Wang Z, Thiruvahindrapuram B, MacDonald JR, Sung WWL, et al. A Comprehensive Workflow for Read Depth-Based Identification of Copy-Number Variation from Whole-Genome Sequence Data. Am J Hum Genet. 2018;102(1):142–55. 29304372 10.1016/j.ajhg.2017.12.007 PMC5777982

[74] Belyeu JR, Nicholas TJ, Pedersen BS, Sasani TA, Havrilla JM, Kravitz SN, et al. SV-plaudit: A cloud-based framework for manually curating thousands of structural variants. GigaScience. 2018;7(7):giy064. 29860504 10.1093/gigascience/giy064 PMC6030999

[75] Robinson JT, Thorvaldsdóttir H, Winckler W, Guttman M, Lander ES, Getz G, et al. Integrative genomics viewer. Nat Biotechnol. 2011;29(1):24–6. 21221095 10.1038/nbt.1754 PMC3346182

[76] Ten Berk de Boer E, Bilgrav Saether K, Eisfeldt J. Discovery of non-reference processed pseudogenes in the Swedish population. Front Genet. 2023;14:1176626. 37323659 10.3389/fgene.2023.1176626 PMC10267823

[77] Feng X, Li H. Higher Rates of Processed Pseudogene Acquisition in Humans and Three Great Apes Revealed by Long-Read Assemblies. Mol Biol Evol. 2021;38(7):2958–66. 33681998 10.1093/molbev/msab062 PMC8661421

[78] Dorshorst B, Henegar C, Liao X, Sällman Almén M, Rubin CJ, Ito S, et al. Dominant Red Coat Color in Holstein Cattle Is Associated with a Missense Mutation in the Coatomer Protein Complex, Subunit Alpha (COPA) Gene. PLoS ONE. 2015;10(6): e0128969. 26042826 10.1371/journal.pone.0128969 PMC4456281

[79] Zhang X, Wacker C, Schütz E, Brenig B. Processed pseudogene confounding the identification of a putative lethal recessive deletion in the bovine 60S ribosomal protein L11 gene (uL5). Anim Genet. 2020;51(1):146–7. 31625165 10.1111/age.12868

[80] Troskie RL, Faulkner GJ, Cheetham SW. Processed pseudogenes: A substrate for evolutionary innovation: Retrotransposition contributes to genome evolution by propagating pseudogene sequences with rich regulatory potential throughout the genome. BioEssays News Rev Mol Cell Dev Biol. 2021;43(11): e2100186. 10.1002/bies.202100186 34569081

[81] Butty AM, Chud TCS, Cardoso DF, Lopes LSF, Miglior F, Schenkel FS, et al. Genome-wide association study between copy number variants and hoof health traits in Holstein dairy cattle. J Dairy Sci. 2021;104(7):8050–61. 33896633 10.3168/jds.2020-19879

[82] Mesbah-Uddin M, Guldbrandtsen B, Lund MS, Boichard D, Sahana G. Joint imputation of whole-genome sequence variants and large chromosomal deletions in cattle. J Dairy Sci. 2019;102(12):11193–206. 31606212 10.3168/jds.2019-16946

