# Supplementary figures and images for: A large structural variant collection in Holstein cattle and associated database for variant discovery, characterization, and application
Source: BMC Genomics. 2024 Sep 30;25:903. doi: 10.1186/s12864-024-10812-2 (PMC11440700; doi:10.1186/s12864-024-10812-2)

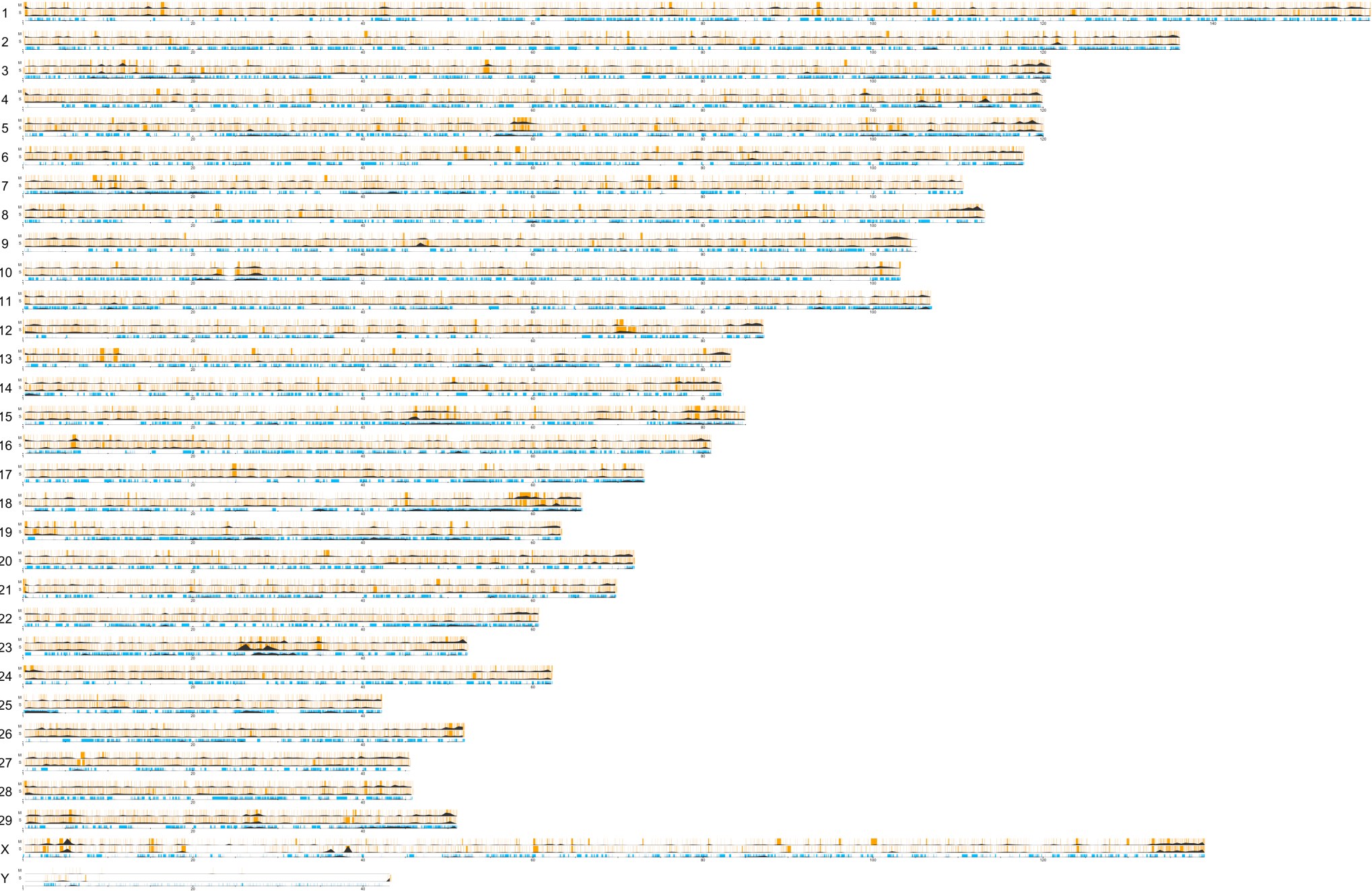

Supplement: Supplementary file 2 — Additional file 2. Distribution of Manta and Smoove SVs and genes in the cattle genome. Each chromosome is represented by three tracks: Manta (top), Smoove (middle) and genes (bottom). The Manta and Smoove tracks show duplications and deletions as orange rectangles and the gene track shows genes as blue rectangles. All tracks are overlaid with density plots of the features in the track using a window size of 500 kb (black). The X-axis is the chromosome position in Mb. The plot was created with the R package karyoploteR. [file 12864_2024_10812_MOESM2_ESM.pdf]

A

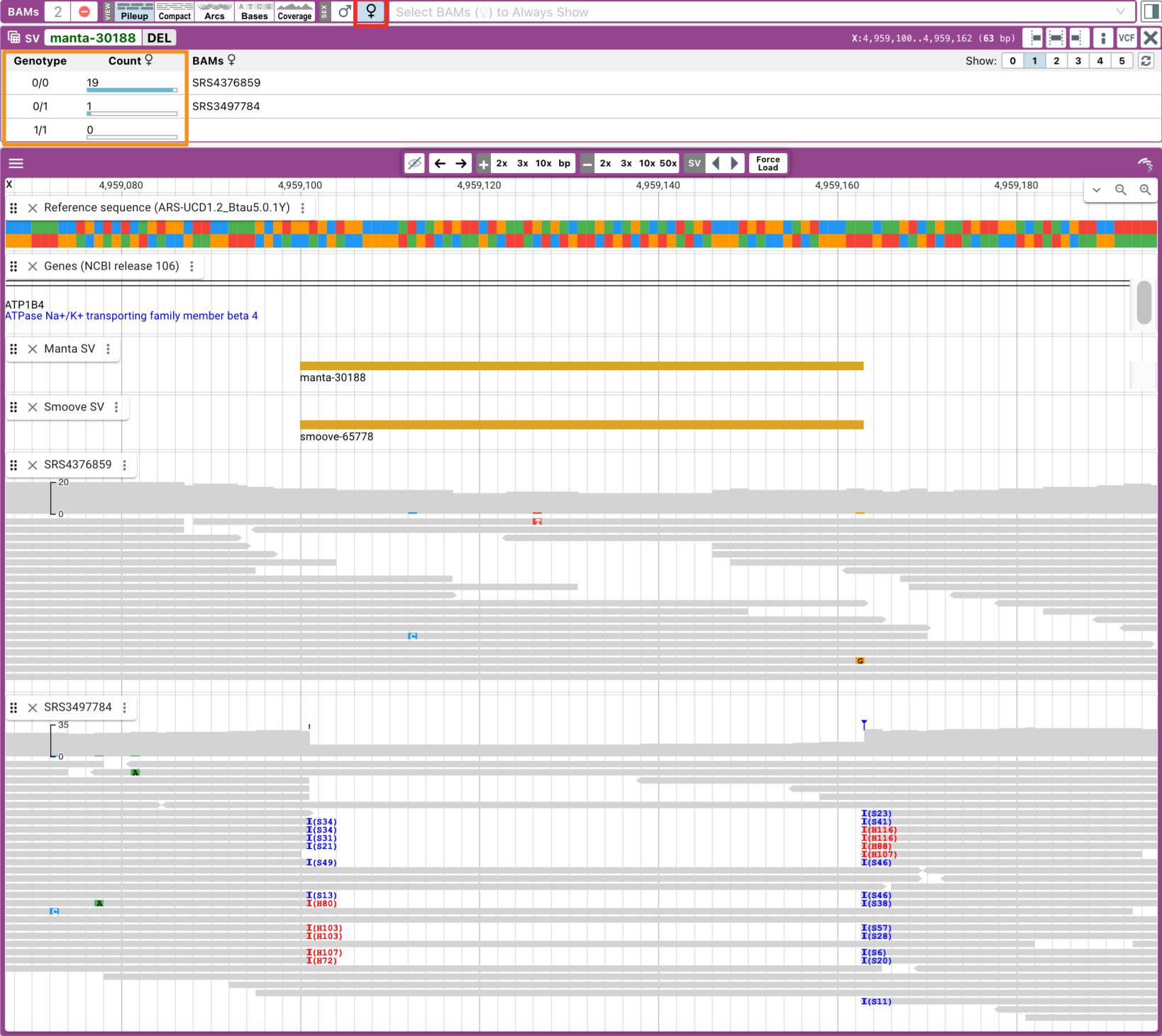

B

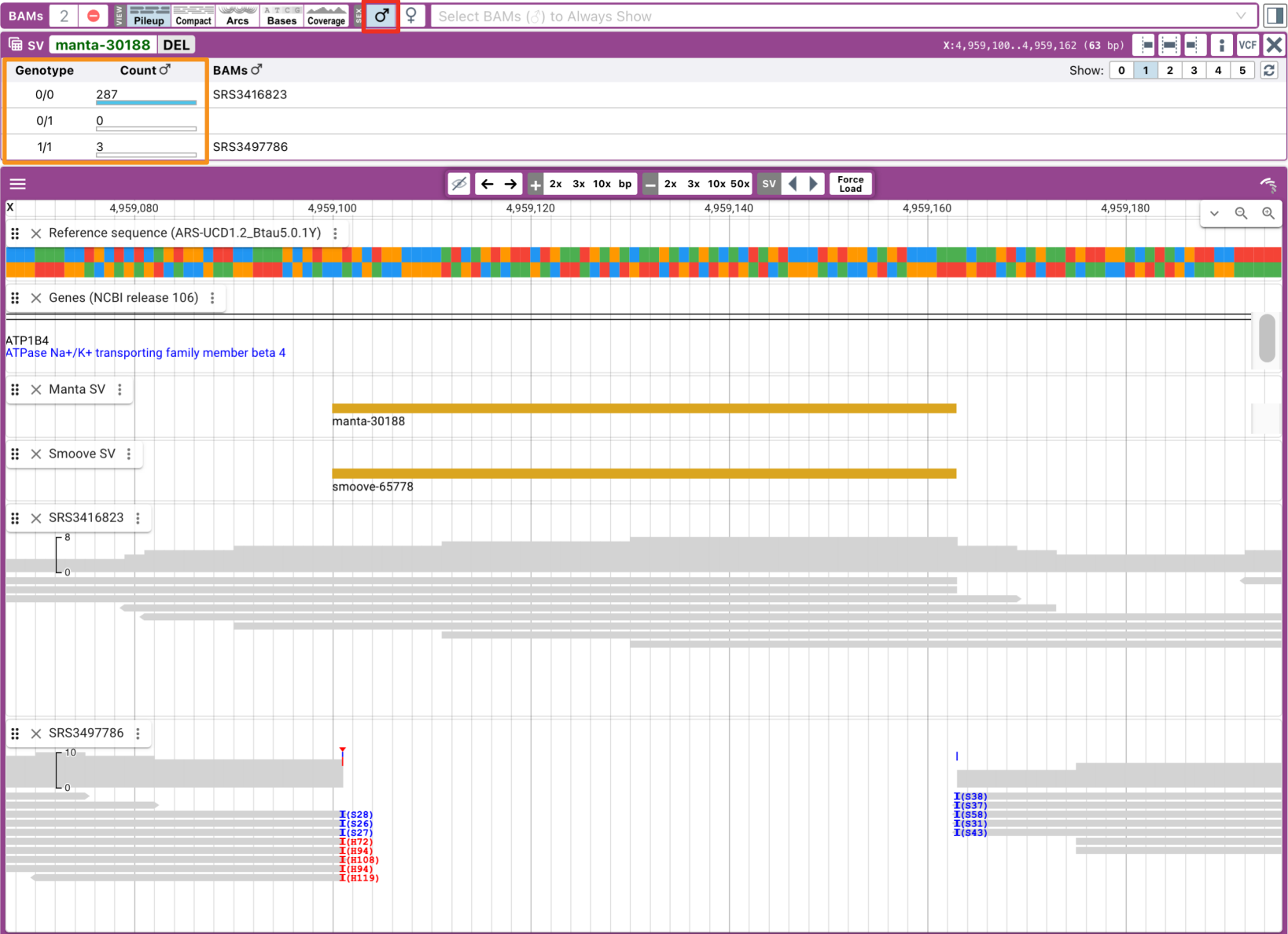

Supplement: Supplementary file 5 — Additional file 5. Sex chromosome SV visualized with SVDB-DC. The manta-30188 / smoove-65778 deletion, located on chromosome X, is shown in (A) female and (B) male samples. The selected sex (red box) restricts the genotype counts (orange box) and the BAM tracks to that sex. The “Genes (NCBI release 106)” track displays gene feature information from NCBI. The two horizontal lines in this track extending the width of the view represent an intron shared by two isoforms (or transcript variants) of the ATP1B4 (ATPase Na+/K+ transporting family member beta 4) gene. The remaining tracks show the Manta and Smoove SVs followed by the read alignment (BAM) tracks for a single sample from each genotype category. [file 12864_2024_10812_MOESM5_ESM.pdf]
